# Reprogrammable, Sustainable, and 3D‐Printable Cellulose Hydroplastic

**DOI:** 10.1002/advs.202402390

**Published:** 2024-05-27

**Authors:** J. Justin Koh, Xue Qi Koh, Jing Yee Chee, Souvik Chakraborty, Si Yin Tee, Danwei Zhang, Szu Cheng Lai, Jayven Chee Chuan Yeo, Jia Wen Jaslin Soh, Peiyu Li, Swee Ching Tan, Warintorn Thitsartarn, Chaobin He

**Affiliations:** ^1^ Institute of Materials Research and Engineering (IMRE) Agency for Science Technology and Research (A*STAR) 2 Fusionopolis Way, Innovis #08‐03 Singapore 138634 Republic of Singapore; ^2^ Institute of High Performance Computing (IHPC) Agency for Science Technology and Research (A*STAR) 1 Fusionopolis Way, Connexis North #16‐16 Singapore 138632 Republic of Singapore; ^3^ Department of Materials Science and Engineering National University of Singapore 9 Engineering Drive 1 Singapore 117575 Republic of Singapore

**Keywords:** 3D‐printing, cellulose, electronics, hydroplastic, sustainability

## Abstract

Modern human societies are highly dependent on plastic materials, however, the bulk of them are non‐renewable commodity plastics that cause pollution problems and consume large amounts of energy for their thermal processing activities. In this article, a sustainable cellulose hydroplastic material and its composites, that can be shaped repeatedly into various 2D/3D geometries using just water are introduced. In the wet state, their high flexibility and ductility make it conducive for the shaping to take place. In the ambient environment, the wet hydroplastic transits spontaneously into rigid materials with its intended shape in a short time of <30 min despite a thickness of hundreds of microns. They also possess humidity resistance and are structurally stable in highly humid environments. Given their excellent mechanical properties, geometry reprogrammability, bio‐based, and biodegradable nature, cellulose hydroplastic poses as a sustainable alternative to traditional plastic materials and even “green” thermoplastics. This article also demonstrates the possibility of 3D‐printing these hydroplastics and the potential of employing them in electronics applications. The demonstrated hydroshapable structural electronic components show capability in performing electronic functions, load‐bearing ability and geometry versatility, which are attractive features for lightweight, customizable and geometry‐unique electronic devices.

## Graphical abstract

In this article, a sustainable cellulose hydroplastic material that can be shaped repeatedly into various 2D/3D geometries using just water is introduced. The bio‐based and biodegradable material possesses excellent mechanical properties comparable to that of commodity plastic and is geometrically reprogrammable. 3D‐printing of the hydroplastic and its application as geometry‐customizable structural electronics material is also demonstrated.

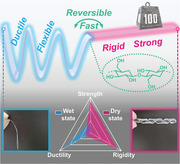

## 1 Introduction

Modern societies are highly dependent on plastic materials, with the main bulk of them being commodity plastics produced in large volumes, such as polyethylene (PE), polypropylene (PP), polyethylene terephthalate (PET) and polyvinyl chloride (PVC). These commodity plastics are petroleum‐based and unsustainable, posing environmental threats when incinerated or pollution when disposed of in landfills and natural environments.^[^ ^1^ ^]^ While their thermoplastic nature provides the convenience of shaping them into desired geometries by softening and molding them at elevated temperatures, these thermal processing techniques, such as injection molding and thermoforming, often require complex machines and large amounts of energy due to the heat and pressure involved during their processing and re‐processing.^[^ ^2^ ^]^

On the other hand, many natural polymers such as cellulose and silk, possess excellent structural properties. However, some of their shortcomings, including processing limitations and hydrophilicity, limit their employment in practical applications.^[^ ^3^ ^]^ Indeed, hydrophilicity leading to water absorption and plasticization effect had also plagued many high‐performance engineering plastics as well, including polymethyl methacrylate (PMMA), polyimides (PI), and polyamides (PA) for decades.^[^ ^4^, ^5^ ^]^ However, the water‐absorbing characteristics of hydrophilic polymers also present opportunities to develop a new sub‐class of polymeric materials, hydroplastics.

In contrast to its thermal analog, hydroplastics can be shaped into various 2D and 3D geometries using just water. For instance, a cellulose derivative, cellulose cinnamate membranes with thickness in the order of tens of microns has been recently reported to possess hydroplastic characteristics.^[^ ^6^ ^]^ In general, hydroplastic materials should consist of a hydratable polymeric network, such that water can penetrate between the polymer chains yet the polymer remains insoluble in an aqueous environment. The plasticization effect of the absorbed water would then provide the hydroplastic with the necessary flexibility and ductility to facilitate the shaping process.^[^ ^7^ ^]^ The absorbed water content should also be of a moderate level so that drying can take place faster and minimize shrinkage upon drying so as to obtain the targeted geometry. This feature sets hydroplastics apart from hydrogels, which can typically contain >90 wt.% of absorbed water content. In the dry state, the hydroplastic should exhibit rigidity similar to that of plastic materials and remain structurally stable in ambient conditions, including in high‐humidity environments.

**Figure 1:**
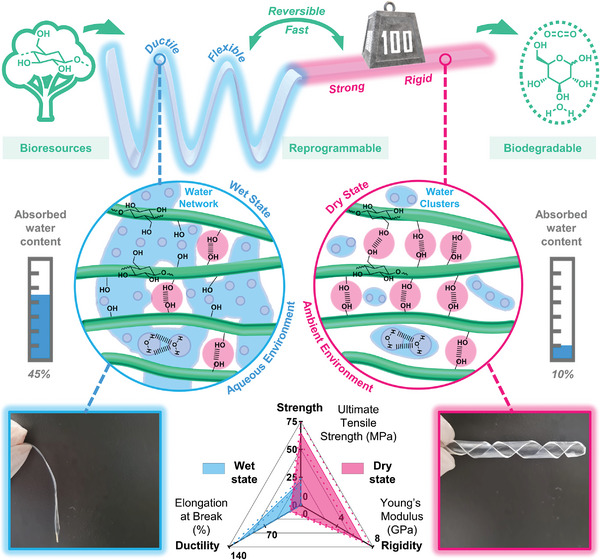
Overview of cellulose hydroplastic. The schematic diagram at the top illustrates the switchable distinctive mechanical behavior of the sustainable cellulose hydroplastic, which is also bio‐based, reprogrammable, and biodegradable. Schematic illustrations in the middle detail the molecular origins of the switchable mechanical behavior of cellulose hydroplastic in its wet and dry state. The radar plot and images at the bottom illustrate the distinctive mechanical properties of cellulose hydroplastic in its wet and dry state.

In this article, a sustainable, reprogrammable and 3D‐printable hydroplastic is introduced in the form of a non‐derivative, non‐fibrous, and highly amorphous cellulose (**Figure** **1**). The cellulose hydroplastic possesses reversible wet and dry states. When submerged in an aqueous environment, the cellulose hydroplastic absorbs a moderate amount of water, forming an interconnected water network that provides the material with high flexibility and ductility. As such, the material can be easily deformed into the desired shape, thereby facilitating the hydroshaping process. The cellulose hydroplastic material can transit into a rigid material with a fixed shape quickly in less than 30 min in an ambient environment (≈60%RH), despite a thickness of hundreds of microns. The rehydration and dehydration process is highly reversible, such that the shape of the material can be easily reprogrammed into other geometries, allowing it to be re‐purposed and thereby extending its application lifetime. The reprogrammability, together with its biodegradability and superior mechanical properties, make cellulose hydroplastic a sustainable alternative to conventional plastic materials. This article also shows the potential of employing cellulose hydroplastic in electronic applications. In particular, 3D‐printed hydroshapable structural electronic components, possessing electronic functions with geometry‐customizability and structural ability were demonstrated.

## 2 Results

### 2.1 Structure of Cellulose Hydroplastic

The cellulose hydroplastic is fabricated via deacetylation of solution‐casted cellulose acetate sheets. It has a chemical structure similar to commercially available α‐cellulose upon complete deacetylation (Figure S1, Supporting Information). Such fabrication route yields a non‐fibrous, non‐derivative cellulose material that is highly amorphous,^[^ ^8^, ^9^ ^]^ with a low crystallinity of 8.6–9.3% (cellulose II polymorph), as characterized by X‐ray diffraction (XRD) (Figure S2, Supporting Information).^[^ ^10^, ^11^ ^]^ Since the amorphous phase of cellulose is known to be water‐accessible,^[^ ^12^ ^]^ the cellulose hydroplastic can contain up to ≈45 wt.% of absorbed water content upon immersion in water, according to thermogravimetric analysis (TGA) (Figure S3, Supporting Information). Dry cellulose hydroplastic equilibrated in 60%RH ambient conditions contains ≈10 wt.% of absorbed water content within its polymeric network (Figure S3, Supporting Information).

### 2.2 Cellulose Hydroplastic Mechanical Behaviour

**Figure 2:**
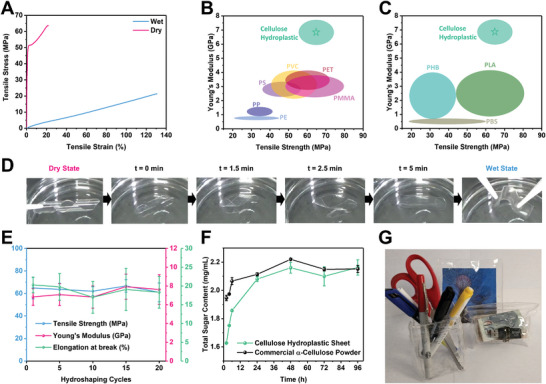
Cellulose hydroplastic as a sustainable alternative. A) Representative stress‐strain curve of cellulose in its wet and ambient (60%RH) dry state. B,C) Ashby plot showing tensile strength and Young's modulus of cellulose hydroplastic in comparison with (B) commodity plastics and with (C) “green” thermoplastics. The star symbol represents the average values and the oval encompasses the standard deviations. D) Snapshots of the rehydration process, taken from Movie S2 (Supporting Information). E) Dry state mechanical properties of cellulose hydroplastic as a function of hydroshaping cycles. F) Total sugar content as a function of time in the enzymolysis solution of cellulose hydroplastic undergoing enzymatic biodegradation. G) Image showing prototypes of hydroshaped commercial products, including a stationery holder, photo frame, and a purse, made from cellulose hydroplastic.

Cellulose hydroplastic exhibits distinctive mechanical behavior in its wet and dry states (Figure 1). In its wet state after immersion in an aqueous medium, it is highly flexible and ductile, possessing a tensile strength of 21.3 ± 2.66 MPa, Young's modulus of 0.0281 ± 0.00180 GPa, and elongation at break of 121.4 ± 13.0%. The flexibility and ductility in the wet state are necessary properties to facilitate the hydro‐shaping process, which would allow the material to undergo significant mechanical deformations to achieve the desired geometry. Ambient equilibrated (at 60%RH) dry cellulose hydroplastic possesses a tensile strength of 64.8 ± 9.51 MPa, Young's modulus of 6.83 ± 0.891 GPa and elongation at break of 20.3 ± 2.10%. Such high strength and elastic modulus, along with moderate ductility, would allow the shaped dry cellulose hydroplastic to operate structurally like a plastic material. The representative stress‐strain curve of the cellulose in the two distinct states is presented in **Figure** **2**A. The mechanical performance of the cellulose hydroplastic in its dry state is superior to most commodity plastics (Figure 2B).^[^ ^13^ ^]^ In fact, it is also superior to many of the popular “green” bio‐based and biodegradable thermoplastic materials,^[^ ^14^ ^]^ such as polylactic acid (PLA), polyhydroxybutyrate (PHB), and polybutylene succinate (PBS) (Figure 2C).^[^ ^15^, ^16^, ^17^, ^18^, ^19^, ^20^ ^]^ Besides, the small amount of absorbed water content (10 wt.%) provides some plasticization effect such that the dry cellulose hydroplastic has a moderate ductility (elongation at break 20.3 ± 2.10%) that many rigid glassy polymers, such as PMMA, PLA, and PHB, do not possess. Therefore, there is a strong prospect for cellulose hydroplastic to serve as a partial solution in resolving global plastic issues through the replacement of unsustainable commodity plastics in many structural applications. Especially with such a high elastic modulus, less volume of the material can be used in structural applications where rigidity is the primary parameter for consideration.

### 2.3 Reprogrammability and Biodegradability

The transition between the two distinct states is highly reversible, making cellulose hydroplastic reprogrammable geometrically. Wet and flexible cellulose hydroplastic can transform into a rigid material with a fixed shape quickly, in only ≈24 min, despite having a thickness in the order of hundreds of microns (Movie S1, Supporting Information). Such a rapid drying mechanism can be partially attributed to the low vaporization enthalpy of water in cellulose originating from the disruption of bulk water structure.^[^ ^14^ ^]^ In addition, a moderate water content unlike typical hydrogels, plays an important part in reducing drying time as well as volumetric shrinkage of the material (Figure S4, Supporting Information). Similarly, dry cellulose hydroplastic can revert back to its flexible and shapable state upon rehydration in an aqueous environment (Figure 2D; Movie S2, Supporting Information). More importantly, it is possible for the mechanical performance of the dry state to remain at a similar level after undergoing multiple hydro‐shaping cycles (Figure 2E; Figure S5, Supporting Information). Therefore, cellulose hydroplastic can be reprogrammed into other geometries for new applications upon reaching their end of life in an application. This would allow them to be re‐purposed which increases their reusability, thereby extending their lifespan and enhancing their sustainable aspect.

Nevertheless, any material would ultimately reach its end of life in practical applications, which is a matter of duration, including the reprogrammable cellulose hydroplastic. In such scenarios, a portion of them would find their way to the natural environment and landfills, leading to the issue of waste pollution and accumulation.^[^ ^21^ ^]^ Therefore, biodegradability is a critical link for materials to truly be sustainable circular materials, which is not always the case for bio‐based materials, such as some bio‐based polyamides. While many cellulose derivatives have been considered non‐biodegradable (e.g. cellulose acetate), cellulose hydroplastic being a non‐derivative has the advantage of biodegradability as pristine cellulose. Using a typical method for biodegradation of cellulose‐based materials with enzymes found in the natural environment,^[^ ^22^, ^23^ ^]^ the increasing release of small and soluble sugar units into the enzymolysis solution indicates its biodegradability (Figure 2F). In fact, cellulose hydroplastic biodegradability is similar to commercial α‐cellulose powder, despite the former being a bulk sheet and the latter being in powdered form with a much larger surface area. This is most probably due to the highly amorphous microstructure of cellulose hydroplastic that is known to be favorable for biodegradation.^[^ ^24^ ^]^ On the other hand, the fragmentation of the cellulose hydroplastic composites (to be discussed in subsequent sections) during the biodegradation process can be easily observed with the presence of light‐absorbing particles.

The fact that cellulose hydroplastic is a bio‐based material already contributes to the reduction of carbon emission upon degradation. Together with its reprogrammable property that would serve to extend the lifespan of the material and its biodegradable nature, cellulose hydroplastic has a strong potential to serve as a sustainable alternative to traditional plastic materials. To demonstrate, some hydroshaped cellulose hydroplastic consumer product prototypes are presented in Figure 2G.

### 2.4 Moisture Resistance of Cellulose Hydroplastic

**Figure 3:**
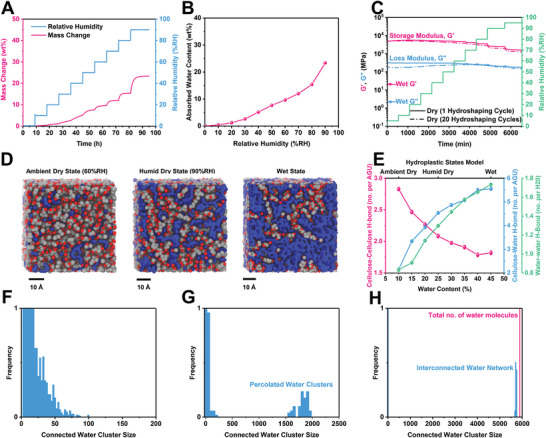
Origins of cellulose hydroplastic state transition. A) Dynamic vapor sorption measurement of cellulose hydroplastic at various relative humidity levels conducted at 25 °C. B) The equilibrated absorbed water content of cellulose hydroplastic at various humidity levels. C) Dynamic mechanical analysis measurement of cellulose hydroplastic showing storage modulus (G’) and loss modulus (G”) of cellulose hydroplastic with increasing relative humidity (RH) from 5 to 95% RH. D) Molecular dynamics simulation box modeling cellulose hydroplastic in the ambient dry state (60%RH), humid dry state (90%RH), and wet state, with 10, 25, and 45 wt.% of absorbed water content respectively. (blue: water molecules, grey: cellulose's carbon atoms, red: cellulose's oxygen atoms). E) Simulated number of hydrogen bonds (H‐bond) for cellulose‐water per anhydroglucose unit (AGU), cellulose–cellulose per AGU, and water‐water per water molecule, as a function of different absorbed water content. F–H) Connected water cluster histogram of simulated amorphous cellulose with (F) 10 wt.%, (G) 25 wt.%, and (H) 45 wt.% absorbed water content.

Given the hydrophilicity of hydroplastics, their resistance to moisture in the ambient environment is definitely a concern. Dynamic vapor sorption (DVS) analysis was conducted (**Figure** **3**A,B) to investigate the amount of absorbed water content at various humidity levels. Indeed, DVS analysis shows that the absorbed water content of dry cellulose at ambient conditions of 60%RH is ≈10 wt.%, in coherence with the TGA measurement discussed in the previous section. With humidity increasing to 80%RH, absorbed water content only increases to ≈15 wt.% of the material. However, with a mere increase in humidity from 80 to 90%RH, absorbed water content increases more steeply to ≈23 wt.%.

Nevertheless, the shaped cellulose hydroplastic remains structurally and geometrically stable even at high humidity levels of 90%RH. In fact, wet hydroplastic can also transit into a rigid state with a fixed shape at a high humidity environment of 90%RH, albeit a much longer time of ≈60 min. Dynamic mechanical analysis at various humidity levels (Figure 3C) shows that the storage modulus (elastic response) is in the order of magnitude of 10^3^ MPa, an order higher than the loss modulus (viscous response) throughout 5–95% RH. Even at high humidity levels of 90 and 95%RH, storage modulus remains in the same order albeit with a relatively steeper drop starting from 80%RH. The viscoelastic properties of the cellulose hydroplastic remain similar even after undergoing 20 hydroshaping cycles. In comparison, the storage modulus and loss modulus of the wet cellulose hydroplastic lie in the order of 10^1^ and 10^0^, respectively, lower than the dry state(s) by two orders of magnitude. All tensile properties of the cellulose hydroplastic, including its Young's modulus which is crucial for cellulose hydroplastic to maintain its rigid structure, remain in the same order of magnitude at 90%RH (Figure S6A, Supporting Information). These findings indicate that the cellulose hydroplastic does indeed possess humidity resistance, such that it remains highly rigid and capable of maintaining its structural integrity even in a humid environment (Figure S6B, Supporting Information).

### 2.5 Origins of Cellulose Hydroplastic State Transitions

In order to further elucidate the molecular origins of the cellulose hydroplastic behavior, the team employed molecular dynamics (MD) simulations to model cellulose hydroplastic with different water content. Figure 3D shows the equilibrated structure of amorphous cellulose with 10, 25, and 45 wt.% of absorbed water content, corresponding to the ambient dry state (60%RH), humid dry state (90%RH), and wet state of the cellulose hydroplastic, respectively.

The hydroxyl groups of cellulose are capable of forming intra/inter‐chain hydrogen bonds (H‐bonding) that are primarily responsible for their high strength and rigidity, but they are also capable of bonding with water molecules.^[^ ^25^ ^]^ Therefore, the amount of water molecules present within the macromolecular network of cellulose hydroplastic would affect its hydrogen bonding structure, and hence, its mechanical behavior.^[^ ^26^, ^27^ ^]^ Generally, higher absorbed water content leads to increased cellulose‐water H‐bonding, along with a reduction of cellulose‐cellulose H‐bonding (Figure 3E). However, above 40 wt.% of absorbed water content, the cellulose–cellulose hydrogen bond appears to saturate. Without further breakage of the cellulose–cellulose hydrogen bond, the cellulose network cannot further expand to accommodate more water molecules, thereby limiting the absorbed water content at a moderate level of 45 wt.% in the wet state. The reduced cellulose–cellulose hydrogen bonding and increased cellulose‐water interaction would provide cellulose with higher chain mobility, leading to a stronger plasticizing effect for cellulose hydroplastic.

However, plasticization alone cannot explain the drastic change in behavior between the humid dry state with 23 wt.% of absorbed water, and the wet state with 45 wt.% of absorbed water. Previous studies have shown that as water content within cellulose increases beyond the percolation of water within cellulose, further increase would lead to a steeper reduction of its stiffness as the water network grows and becomes more interconnected.^[^ ^28^ ^]^ In the ambient dry state with 10 wt.% water content, the water–water hydrogen bond per water molecule is <1, at 0.833 ± 0.02, with water molecules existing as isolated clusters of typically <80 molecules (Figure 3F; Figure S7A, Supporting Information). However, the appearance of percolated clusters begins to appear at 20 wt.% water content (Figure S7C, Supporting Information). This explains the increasingly steeper reduction in elastic modulus starting from 80%RH (Figure 3C), where absorbed water content crosses 20 wt.% at 90%RH. Fortunately, at a high humidity environment of 90%RH, it is still at the beginning of the drastic drop in rigidity for cellulose hydroplastic, allowing cellulose hydroplastic to maintain its structural integrity. Indeed, with 45 wt.% of absorbed water content in the wet state, the water–water hydrogen bond per water molecule reaches 1.73 ± 0.01, with ≈97% of the water molecules participating in a single interconnected water network (Figure 3H; Figure S7F, Supporting Information). This explains the distinct flexibility and ductility of the cellulose hydroplastic in its wet state, as compared to the dry state(s). The MD results on water connectivity are also in coherence with Raman scattering spectroscopy analysis (Figure S8, Supporting Information).

### 2.6 Cellulose–Carbon Hydroplastic Composites

**Figure 4:**
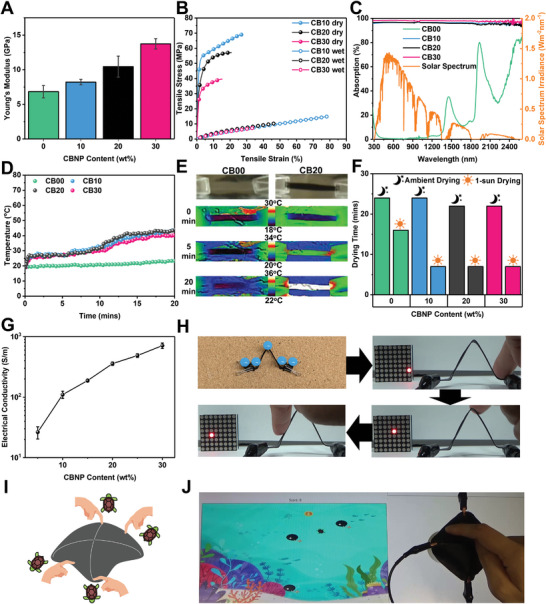
Cellulose‐carbon hydroplastic composites. A) Young's modulus of cellulose‐carbon hydroplastic composites with various carbon nanoparticles (CBNPs) loading. B) Representative stress‐strain curves of cellulose‐carbon hydroplastic composites in both wet and dry state. C) Absorption spectra of cellulose‐carbon hydroplastic composites at different wavelengths. D) Surface temperature of cellulose–carbon hydroplastic composites under 1‐sun illumination as a function of time. E) Thermal images of CB00 and CB20 at different time periods under 1‐sun illumination. F) Drying time of cellulose‐carbon hydroplastic composites in a room ambient environment and under 1‐sun illumination. G) Electrical conductivity of cellulose‐carbon hydroplastic composites as a function of CBNPs loading. H) Demonstration of the “corkboard method” to hydroshape a CB20 strip for a capacitive sensor. I) Schematic illustration of dome‐shaped CB20 capacitive sensor with four quadrants controlling in‐game turtle movement. J) In‐game image of CB20 capacitive sensor.

Unlike most cellulose‐based polymer matrix composites where fibrous/particulate cellulose is the reinforcing filler,^[^ ^29^, ^30^, ^31^ ^]^ functional fillers can be easily incorporated into the cellulose hydroplastic matrix. To demonstrate the versatility of fabricating hydroplastic composites with enhanced performance, up to 30 wt.% carbon black nanoparticles (CBNPs) are incorporated into cellulose hydroplastic (Figure S9, Supporting Information). Beyond 30 wt.% CBNPs loading, the samples are too brittle for the fabrication and hydroshaping process. The cellulose‐carbon composites are denoted as CBXX (XX represents the CBNPs content in wt.%). Mechanically, CBNPs serve as reinforcement for the cellulose matrix. The Young's modulus increases with increasing CBNPs loading (**Figure** **4**A), indicating effective stress transfer in the elastic region. With 30 wt.% CBNPs loading, CB30 achieves Young's modulus of ≈14 GPa. Meanwhile, the hydroplastic characteristics of the cellulose‐carbon composites are retained, whereby all the wet composites exhibit similar flexibility and relatively higher ductility, as shown in their typical stress‐strain curve presented in Figure 4B. Details of their mechanical properties can be found in Table S1 (Supporting Information). In addition, the composites show similar biodegradability as the pristine cellulose hydroplastic. They appear to fragment more easily with higher CBNPs content under similar enzymatic biodegradation conditions, possibly due to the increased surface for degradation that arises from the interface between the cellulose matrix and the CBNPs fillers (Figure S10, Supporting Information). The black residues collected after biodegradation were characterized to be identical to the pristine CBNPs, providing a sustainable pathway to recycle precious fillers incorporated into the cellulose hydroplastic (Figure S11, Supporting Information).

The optical properties of the composites are also augmented. Pristine cellulose hydroplastic is highly transparent due to its amorphous nature, having a transmittance of >95% within the visible light range (Figure S12, Supporting Information). With CBNPs acting as light absorbers, all the cellulose‐carbon composites exhibit absorption of >95% in most of the entire range of ultraviolet‐visible‐infrared wavelengths between 260–2600 nm, as presented in Figure 4C. This feature can be exploited for photothermal effect to accelerate the drying process. The effectiveness of the photothermal drying strategy of the composites with similar thickness was evaluated using 1‐sun irradiation. Upon 1 sun irradiation, the composites with CBNPs embedded experience a rapid rise in surface temperature from ≈19 to ≈27 °C in less than a minute (Figure 4D). This is followed by a steady‐state region with temperature maintained at ≈27 °C due to the endothermic process of water vaporization. Upon eliminating a large portion of water after ≈6 min of solar irradiation, the temperature of the composites starts to rise rapidly again, reaching ≈40 °C after 20 min of exposure to 1‐sun irradiation. In contrast, pristine cellulose hydroplastic (i.e., CB00) exhibits only a very modest linear increase in surface temperature, reaching only 23 °C after 20 min. The thermal images at 0, 5, and 20 min are shown in Figure 4E. The effectiveness of photothermal drying of the various hydroplastic materials is summarized in Figure 4F, which shows the time taken to transit from a wet and flexible state into a shape‐fixed rigid state. In ambient conditions, the time taken for composite materials to dry does not differ much from pristine cellulose, at ≈23–24 min. Under 1‐sun irradiation, the drying time of the composite with CBNPs is shortened to only ≈7 min, a third of the pristine cellulose hydroplastic. Since light sources are readily available in both industrial and household settings, photothermal enhanced quick drying is an attractive feature for mass production and household utilization of hydroshaped products.

The cellulose–carbon composites also exhibit higher electrical conductivity with increasing CBNPs loading (Figure 4G). In addition, a free‐forming hydroshaping technique that uses pins and a corkboard coined the “corkboard method”, is introduced (Figure 4H). This would allow hydroplastics to be shaped without the need of fixed molds, and be easily executed even in the household environment. To show the applicability of these conductive hydroplastic composites in electronics applications, strips of CB20 were hydro‐shaped using the corkboard method and demonstrated their touch sensor abilities. Through the employment of a surface‐capacitive system with lock‐in amplification (i.e., phase‐sensitive detection) to measure the small impedance change of the sensor induced by the body capacitance (Figure S13, Supporting Information),^[^ ^32^ ^]^ the inverted V‐shaped sensor can detect various touch positions as shown in Figure 4H (Movie S3, Supporting Information). The versatility of this shaping technique allows sensors of various shapes to be easily fabricated (Movie S4, Supporting Information). Similarly, sheet‐like sensors can be shaped into 3D structures. Figure 4I illustrates a dome‐shaped sensor with four defined quadrants capable of sensing various touch positions (see Figure S14, Supporting Information). The high conductivity of the sensor allows the body impedance to be measured quickly due to the high bandwidth of the sensing circuit, allowing the impedance measurement to be translated into touch positions in real‐time. This enables its use as a touch sensor in applications requiring fast responses of >30 Hz, such as a game controller (Figure 4J; Movie S5). Unlike soft and conformable sensors that would require a rigid structure at the base to support and provide a shape to these sensors,^[^ ^33^, ^34^ ^]^ hydroplastic sensors can be shaped into a desired shape and possess sufficient rigidity and strength to withstand mechanical forces while maintaining their structural integrity. This opens up the possibility of employing hydroplastic composites for structural electronics, where the multifunctional materials perform both electronic and load‐bearing functions, resulting in material‐saving and weight‐reducing electronic devices.

### 2.7 3D‐Printing of Hydroshapable Electronics

**Figure 5:**
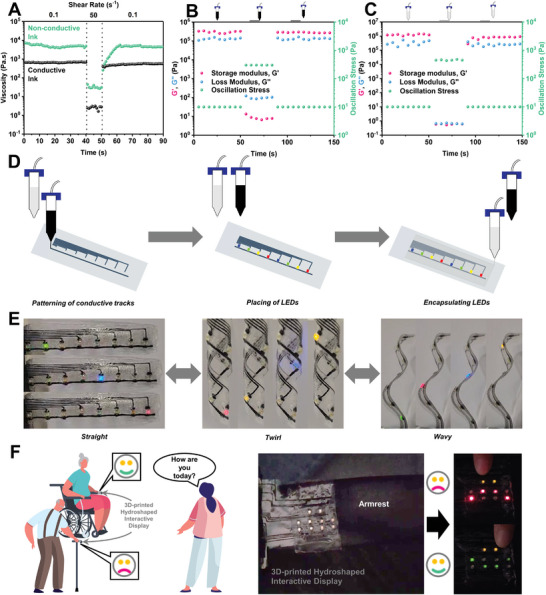
3D‐printed hydroshapable electronics. A) The viscosity of conductive (CB30) and non‐conductive (CB00) inks as a function of three shear rate steps (low, high, low). B,C) Storage modulus (G′) and loss modulus (G″) as a function of three oscillation stress steps (<flow stress, >flow stress, <flow stress) for (B) conductive ink and (C) non‐conductive ink. D) Schematic illustration of 3D‐printing a hydroplastic PCB. E) Demonstration of the hydroplastic PCB's reprogrammability into various geometrical shapes. F) 3D‐printed hydroshapable interactive display incorporated with resistive sensors and LED displays for non‐verbal communications.

3D‐printing brings many advantageous features to electronics that are otherwise unattainable by traditional manufacturing.^[^ ^35^ ^]^ The cellulose hydroplastic and its composites can be 3D‐printed using an extrusion‐based direct‐ink‐writing technique.^[^ ^36^ ^]^ Two inks were formulated with the aid of a non‐toxic ethyl acetate solvent: one conductive (CB30) and the other non‐conductive (CB00). As shown in the step‐shear rate measurement in **Figure** **5**A, both inks exhibit a shear‐thinning behavior, whereby an increase in shear rate would lead to a reduction in viscosity. However, the two inks responded differently, upon the reverse of the shear rate from high to low. The conductive ink would return to its original viscosity almost instantaneously, while the recovery for the non‐conductive ink is time‐dependent due to its thixotropic nature. For step‐stress measurements (Figure 5B,C), both inks exhibit an elastic response (G′) dominance at low shear stress, indicating a solid‐like behavior. With shear stress being increased to a level above their flow stress (Figure S15), the ink behavior would transit toward viscous response (G″) dominating, which would allow the ink to be extruded out of the printhead nozzle. Upon switching back to low shear stress, the conductive ink exhibits a quick recovery (Figure 5B), while the non‐conductive ink exhibits a similar time‐dependent recovery with the viscous response dominating over the elastic response at the initial period before the elastic response recovers to a pre‐shear level (Figure 5C).

To demonstrate 3D‐printed hydroshapable electronic components, the two inks were employed to fabricate a printed circuit board (PCB) as illustrated in Figure 5D. The conductive ink can be printed onto the non‐conductive material to create conductive tracks with customized circuit designs. The quick recovery nature of the conductive ink would allow fine lines to be created without the extruded material spreading over a large area. Although some printable conductive polymers can also be employed for such purposes, they typically lack the hydroshapable capability, and their compatibility with the hydroplastic substrate is a concern (Figure S16, Supporting Information). Following the printing of the circuit, the non‐conductive ink is then used to encapsulate the LEDs and the printed circuits. The thixotropic nature of the ink would then allow adjacent extruded struts to impinge upon each other and eventually heal for a smooth and continuous surface.

Modern‐day electronics, including wearables, come in a wide variety of geometries. However, many of their geometries are still restricted by their rigid internal components, for instance, traditional PCBs have conductive tracks patterned onto rigid materials. More advanced flexible PCBs are also available in the market, capable of conforming to some geometries with applied stress. Here, a green, sustainable, and hydropshapable PCB is introduced. The green hydroplastic PCB can be repeatedly hydroshaped into various shapes, including sheet, twirl, and wavy shapes, as illustrated by the LED display in Figure 5E (Movie S6, Supporting Information). They can be easily shaped into geometries that accommodate the electronic device without applied stress, and at the same time provide load‐bearing/structural properties to the device. This would provide electronics with more versatility in terms of geometries and possibly achieve a more compact design.

In addition, resistive buttons can also be printed by leaving a small 1 mm gap between the conductive tracks, which can serve as sensors for digital inputs by humans. For instance, an electric *Xun* with its holes replaced by these digital resistive buttons, and musical notes programmed to be played with various fingering positions modeled after a classical 6‐hole Xun is demonstrated (Figure S17, Table S2, and Movie S7, Supporting Information). Combining these resistive buttons with the aforementioned LED display would then create a 3D‐printed hydroshapable interactive display (Figure S18, Supporting Information). Such interactive displays can be easily shaped and incorporated geometrically with different structures. To demonstrate, an interactive display that can be hydroshaped onto the armrest of a wheelchair or walking stick was fabricated. Such devices can serve as a form of non‐verbal communication for individuals with conditions such as oromotor dysfunctions (Figure 5F) (Movie S8, Supporting Information).

## 3 Discussion

In summary, highly amorphous cellulose is introduced as a sustainable and environmentally friendly hydroplastic material, that can be shaped repeatedly into various 2D/3D geometries using just water. Cellulose hydroplastic absorbs a moderate amount of water in an aqueous environment but remains insoluble, leading to a plasticization effect that provides the necessary flexibility and ductility for the hydroshaping process. Wet cellulose hydroplastic can transit into a rigid state with a fixed shape in <30 min in ambient conditions (60%RH). In the dry state, it exhibits high strength and high stiffness, which are superior to most commodity plastics and “green” bio‐based, biodegradable thermoplastic materials. Despite its hydrophilicity, it is capable of maintaining its rigid shape even in humid environments (90%RH). The rigid dry and flexible wet states are highly reversible, allowing cellulose hydroplastic to be geometrically reprogrammed repeatedly, which allows them to be repurposed. Given its bio‐based, biodegradable, and reprogrammable nature, along with its excellent mechanical properties, cellulose hydroplastic is a sustainable circular material that has the potential to be a partial solution for commodity plastics replacement in applications that do not require direct contact with water, reducing the reliance on non‐renewable petroleum‐based plastics, the energy consumed used for thermal processing and mitigating the plastic pollution issue at the same time.

Cellulose hydroplastic can also be incorporated with functional filler to be fabricated into multifunctional composite materials to improve its hydroshaping process or expand its applicability. For example, this was demonstrated through the incorporation of CBNPs, providing the hydroplastic composite with mechanical reinforcement, photoadsorption and electrical conductivity. In particular, photo‐absorption opens up the possibility for photo‐enhanced drying of cellulose hydroplastic to hasten the hydroshaping process. More importantly, this article demonstrated the applicability of cellulose hydroplastic for electronics applications. For instance, hydroshapable capacitive sensors were demonstrated. The cellulose hydroplastics and its composites are also 3D‐printable. For example, a 3D‐printed hydroshapable interactive display with both LED displays and resistive digital sensors was demonstrated, by printing a combination of both electrically conductive and non‐conductive hydroplastic materials. These hydroshapable electronic components exhibit geometry customizability, load‐bearing abilities and perform electronics functions at the same time, which are advantageous for lightweight, compact, and geometry‐unique electronic devices. This work serves as the foundation for future development and employment of hydroplastic materials in structural‐functional applications.

## 4 Experimental Section

### Materials and Chemicals

Cellulose acetate (Mn: 50,000 g mol^−1^, acetyl content: 39.7wt.%, Sigma‐Aldrich), sodium carboxymethyl cellulose (Mw: ≈250 000, degree of substitution: 0.7, Sigma‐Aldrich), α‐cellulose (powder, Sigma‐Aldrich), sodium hydroxide (purity: 99%, Sigma Aldrich), sodium acetate (purity: >99%, Sigma‐Aldrich), acetic acid (purity: >99%, Sigma‐Aldrich), methanol (purity: 99.8%, J.T. Baker), ethyl acetate (purity: 99.9%, J.T. Baker), Anthrone (purity: 97%, Sigma Aldrich), cellulase (from *Aspergillus niger*, activity: ≥0.3 units mg^−1^, Sigma‐Aldrich), *n*‐butyl alcohol (purity: >99%, Sigma‐Aldrich) carbon black nanoparticles (Super C65 TIMCAL) were used as received.

### Fabrication of Cellulose Hydroplastic and Composites

Cellulose acetate powder (5 wt.%) was dissolved in ethyl acetate and cast onto PTFE dishes. Evaporation of ethyl acetate would lead to the formation of a cellulose acetate film of ≈0.3 mm thick. Subsequent deacetylation of the cellulose acetate film would take place in 0.2 mol L^−1^ NaOH/methanol solution for >3 h. The obtained cellulose hydroplastic film was then rinsed in water and soaked for at least 1 day. Similarly, for the cellulose–carbon hydroplastic composite, CBNPs were dispersed into the ethyl acetate solution prior to the dissolution of cellulose acetate. The drying and hydro‐pshaping process typically takes place in the ambient environment of 60%RH, unless otherwise stated.

### Biodegradation

The biodegradability of the materials was evaluated by in vitro enzymatic hydrolysis by cellulase, similar to previous reports.^[^ ^22^, ^23^ ^]^ Briefly, the enzyme solution was formulated by adding 0.5 g of cellulase (≥0.3 units mg^−1^) to 100 mL of HAc/NaAc buffer solution of pH 4.8. Thin sheets of cellulose hydroplastic and its composites, of ≈8 mg, were added into the enzyme solution, and heated to 50 °C in a water bath. At various time intervals, 1 mL of the enzymolysis solution was extracted and deproteinized with 200 µL of sevage reagent that consisted of chloroform and n‐butyl alcohol with a volume ratio of 5:1. To measure the total sugar content, 200 µL of the deproteinized enzymolysis solution were added to 800 µL of anthrone sulfate solution, followed by incubation in boiling water bath for 10 min. The absorbance of the final mixture at 620 nm was then measured using a microplate reader (Tecan, Infinite M200). The total sugar content of the enzymolysis solution was then determined by mapping the absorbance with a standard glucose curve.

### Material Characterizations

For mechanical testing, the hydroplastic samples were cut in dogbone specimens according to ASTM D638 type V standard with 7.62 cm (gauge length) × 3.18 cm (width). Tensile tests were conducted using an Instron 5569 universal tensile machine, with a load cell of 1 kN and crosshead speed of 5 mm min^−1^. A total of 5 specimens were tested for each sample type. The dried samples were stabilized in ambient conditions (≈60% RH) for at least a week prior to mechanical testing.

The water content of the wet and ambient dried cellulose hydroplastic was measured using thermogravimetric analysis, Q500 by TA Instruments. The wet hydroplastic samples were wiped dry using fibrous cellulose paper before testing to remove surface water. The ramp rate for the temperature was set at 3 °C min^−1^. Dynamic vapor sorption of the dry state cellulose hydroplastic was conducted using Aquadyne DVS at 25 °C to obtain the absorbed water content at various humidity levels. The water absorption isotherms were obtained after equilibrating for 9 h at relative humidity levels except for 90%RH, which was equilibrated for 14 h.

Dynamic mechanical analysis with a humidity chamber, TA instrument Q800, was used to measure the viscoelastic properties of cellulose hydroplastic at various humidity levels and temperatures. Film tension mode with an amplitude of 20 µm and frequency of 1 Hz was applied for the measurements. A humidity sweep was conducted at 25 °C condition. Similar to the equilibration of dynamic vapor sorption, starting from 5%RH, every step increase in relative humidity levels was equilibrated for 9 h, except for 90 and 95%RH, which were equilibrated for 14 h each. For the temperature sweep, the ramp rate was at 3 °C min^−1^ from 25 to 100 °C.

The ATR‐FTIR measurements were performed using an Agilent Cary 660 spectrometer with Pike Gladi ATR. X‐ray diffraction was characterized using an X‐ray Bruker, D8‐Advance X‐ray diffractometer, with Cu Kα_1_ radiation of 1.54056 Å. Raman scattering spectroscopy measurements were obtained using Renishaw InVia Raman microscope with 532 nm green laser, 10% of laser power, 10s exposure time, and 2 accumulations between wavenumber of 2800–3000 cm^−1^. The absorption and transmittance of the hydroplastics were measured using Shimadzu UV–vis–NIR Spectrometer, UV‐3600, with an integrating sphere. Transmission electron microscopy (TEM) images were taken on a Tecnai X‐TWIN transmission electron microscope with an acceleration voltage of 200 kV. Scanning electron microscopy (SEM) images were characterized by a JEOL JSM7600F field‐emission scanning electron microscope (FESEM), with an accelerating voltage of 5 kV. The electrical conductivity of the cellulose–carbon hydroplastic composites was measured using a four‐point probe by Nittoseiko Analytech with a probe distance of 1.5 mm.^[^ ^37^ ^]^

### Molecular Dynamics Simulation

Amorphous cellulose models were configured from the cellulose builder tool.^[^ ^38^ ^]^ Each cellulose chain is an I‐beta with a 20‐mer chain length. The CHARMM C36 carbohydrate force field was adopted.^[^ ^39^, ^40^ ^]^ Simulation models have 40 cellulose chains in the box. Water molecules, with numbers varying from 800 (dry state) to 5891 (wet state), were inserted randomly. A 2 fs time step and periodic boundary condition were used. Particle Mesh Ewald (PME) algorithm was applied along with a 12 Å cut‐off for the neighbor search and real‐space electrostatics;^[^ ^41^, ^42^ ^]^ van der Waals cut‐off was 12 Å. The V‐rescale thermostat (τ = 0.1 fs) and Parrinello‐Rahman barostat (τ = 1 fs) were used to equilibrate the systems.^[^ ^43^, ^44^ ^]^ After simulated annealing, the systems were simulated for a total of 20 ns at atmospheric pressure and 300 K with an NPT ensemble. For hydrogen bond analysis, the geometric criteria for the distance threshold (donor–acceptor) are 0.35 nm, and the cut‐off angle cut‐off is 50° (hydrogen‐donor–acceptor). The last 1 ns of equilibrated trajectory was used to perform the analysis.

### Ambient and Photothermal Drying

Rectangle strips of cellulose hydroplastic and cellulose‐carbon hydroplastic composites with the dimensions 40 mm × 5 mm × 0.3 mm (L × W × T) were cut out for drying test. Drying time is determined to be from the time when the strips are wiped dry externally after being removed from the aqueous medium to the time when they become rigid with a fixed shape. Ambient drying takes place in a 60% RH environment. Photothermal drying takes place in a similar environment but with an additional projection of photoirradiation with an intensity equivalent to AM 1.5 G condition (100 mW cm^−2^) by a solar simulator (Yamashita Denso, Model: YSS‐80S). A thermal camera (HIKMICRO M30) was used to take thermal images and measure the surface temperature of the materials during the drying process.

### Hydroplastic Capacitive Touch Sensor

Strip and sheet CB20 were used for the capacitive touch sensor demonstration. Their circuit schematics can be found in Figures S13 and S14 (Supporting Information), respectively. Capacitance changes induced by the human body capacitance are measured by using op amps (Analog Devices AD8065) to convert a small AC current signal to a voltage measured by SR830 lock‐in amplifiers.

### 3D‐Printing of Electronic Components

Two inks were formulated for the 3D printing of electronic components. The thixotropic non‐conductive ink was formulated with 20 wt.% of cellulose acetate in ethyl acetate, and the non‐thixotropic conductive ink was formulated with 16.8 wt.% of cellulose acetate and 7.2 wt.% of CBNPs in ethyl acetate.

Rheology studies of the inks were conducted using a TA Instruments discovery series rotational rheometer with a 40 mm parallel plate and 500 µm gap. The step‐shear rate measurements were conducted in three steps, starting with a low shear rate (0.1 s^−1^) for 40 s, followed by a high shear rate (50 s^−1^) for 10 s, and then back to a low shear rate (0.1 s^−1^) for 40 s. Amplitude sweep measurements were conducted at a constant angular frequency of 6.283 rad s^−1^ to determine the flow stress of the ink. Similarly, the step‐stress measurements were also conducted with a constant angular frequency of 6.283 rad/s in three steps. A low stress below the flow stress (10^1^ Pa) of the inks is applied in the first and third steps, whereas a stress higher than the pre‐determined flow stress of the inks is applied in the 2nd step.

Printing of the PCB and resistive sensor was carried out using a Cellink BIOX bioprinter with three printheads. The conductive and non‐conductive inks were loaded into a 3 mL syringe on separate pneumatic printheads. They were printed using 27G and 25G tapered dispensing tips, with 150–250 kPa and 250–350 kPa air pressure, respectively. Typical print speed is 5–8 mm s^−1^. The printed components were left in the ambient to dry out the ethyl acetate prior to the deacetylation process.

## Conflict of Interest

The authors declare no conflict of interest.

## Supporting information

Supporting Information

Supplemental Movie 1

Supplemental Movie 2

Supplemental Movie 3

Supplemental Movie 4

Supplemental Movie 5

Supplemental Movie 6

Supplemental Movie 7

Supplemental Movie 8

## Data Availability

The data that support the findings of this study are available from the corresponding author upon reasonable request.
