# Supplementary material for: Reprogrammable, Sustainable, and 3D‐Printable Cellulose Hydroplastic
Source: Adv Sci (Weinh). 2024 May 27;11(29):2402390. doi: 10.1002/advs.202402390 (PMC11304289; doi:10.1002/advs.202402390)
Supplement: Supplementary file 1 — Supporting Information [file ADVS-11-2402390-s004.pdf]

ADVANCED SCIENCE
Open Access

Supporting Information

for *Adv. Sci.*, DOI 10.1002/advs.202402390

Reprogrammable, Sustainable, and 3D-Printable Cellulose Hydroplastic

J. Justin Koh, Xue Qi Koh, Jing Yee Chee, Souvik Chakraborty, Si Yin Tee, Danwei Zhang, Szu Cheng Lai, Jayven Chee Chuan Yeo, Jia Wen Jaslin Soh, Peiyu Li, Swee Ching Tan, Warintorn Thitsartarn and Chaobin He**

Supplementary Information

Reprogrammable, Sustainable and 3D-printable Cellulose Hydroplastic

J. Justin Koh[a], Xue Qi Koh[a], Jing Yee Chee[a], Souvik Chakraborty[b], Si Yin Tee[a], Danwei Zhang[a], Szu Cheng Lai[a], Jayven Chee Chuan Yeo[a], Jia Wen Jaslin Soh[a,c], Peiyu Li[a,c], Swee Ching Tan[c], Warintorn Thitsartarn[a], Chaobin He[a,c]**

[a]Institute of Materials Research and Engineering (IMRE), Agency for Science, Technology and Research (A*STAR), 2 Fusionopolis Way, Innovis #08-03, Singapore 138634, Republic of Singapore.

[b]Institute of High Performance Computing (IHPC), Agency for Science, Technology and Research (A*STAR), 1 Fusionopolis Way, Connexis North #16-16, Singapore 138632, Republic of Singapore.

[c]Department of Materials Science and Engineering, National University of Singapore, 9 Engineering Drive 1, Singapore 117575, Republic of Singapore.

Corresponding Authors: Dr. J. Justin Koh (email: justin_koh@imre.a-star.edu.sg); Prof. Chaobin He (email: msehc@nus.edu.sg)

Supplementary Figures

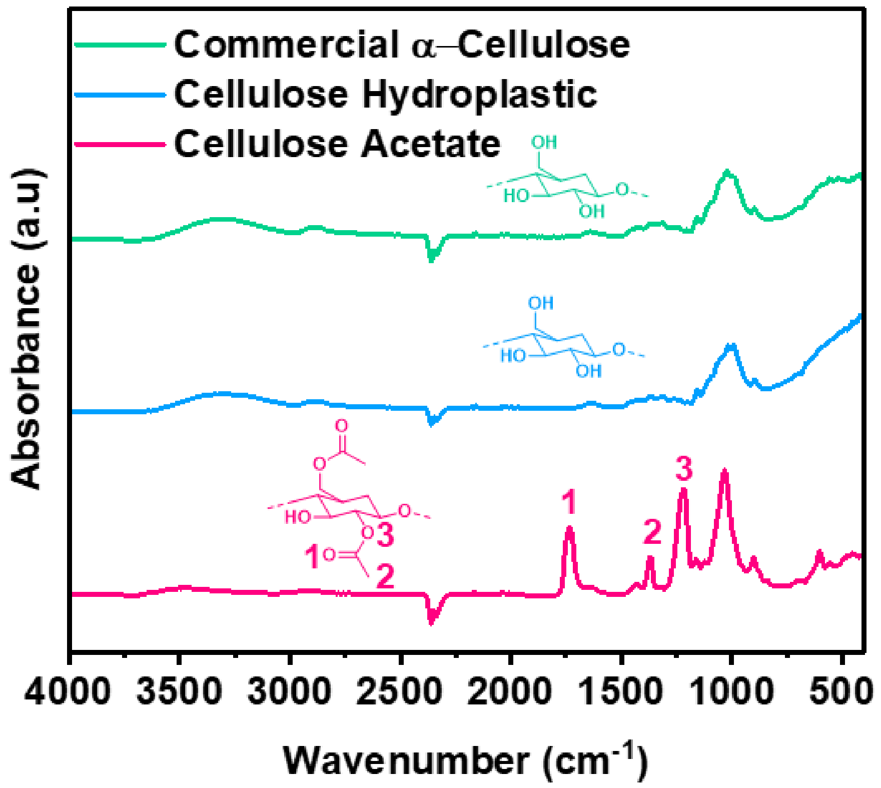

Figure S1. ATR-FTIR spectroscopy of cellulose hydroplastic, cellulose acetate and commercial α-cellulose.

Cellulose hydroplastics have chemical structure similar as pristine α-cellulose upon successful deacetylation of cellulose acetate sheets, with the disappearance of the three distinct peaks at 1735 cm^{-1}, 1370 cm^{-1} and 1220 cm^{-1}, corresponding to the carbonyl bond stretching (νC=O), methyl bending (δC-CH_3) and alkoxy group stretching (νC-O-C) vibrations of the acetate group, respectively.

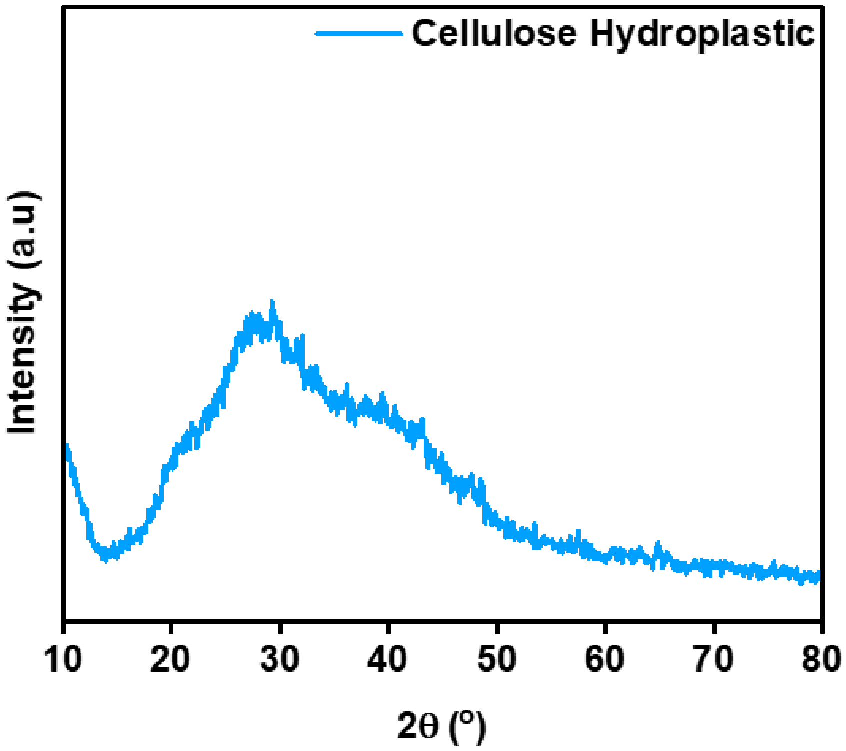

Figure S2. X-ray diffraction (XRD) showing highly amorphous nature of cellulose hydroplastic.

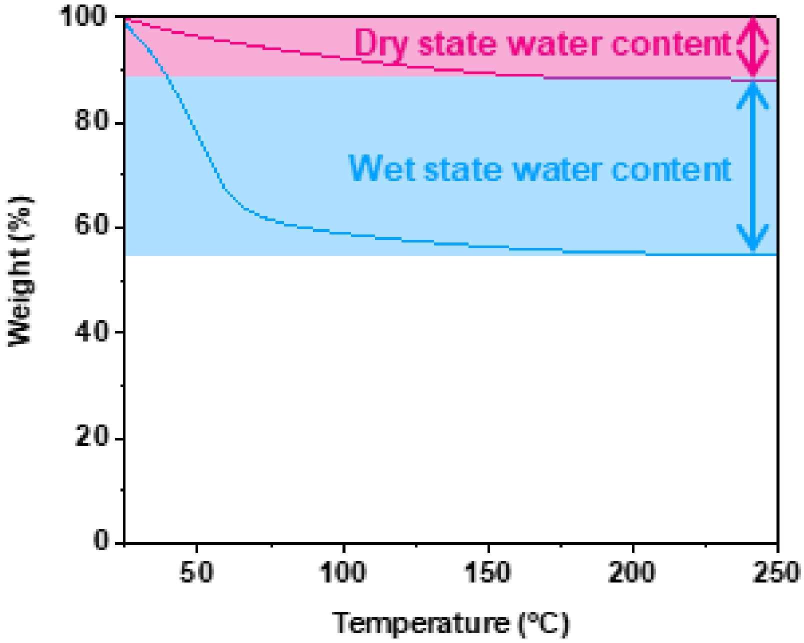

Figure S3. Water content measurement of cellulose hydroplastic in wet state and ambient (60%RH) dry state by thermogravimetric analysis.

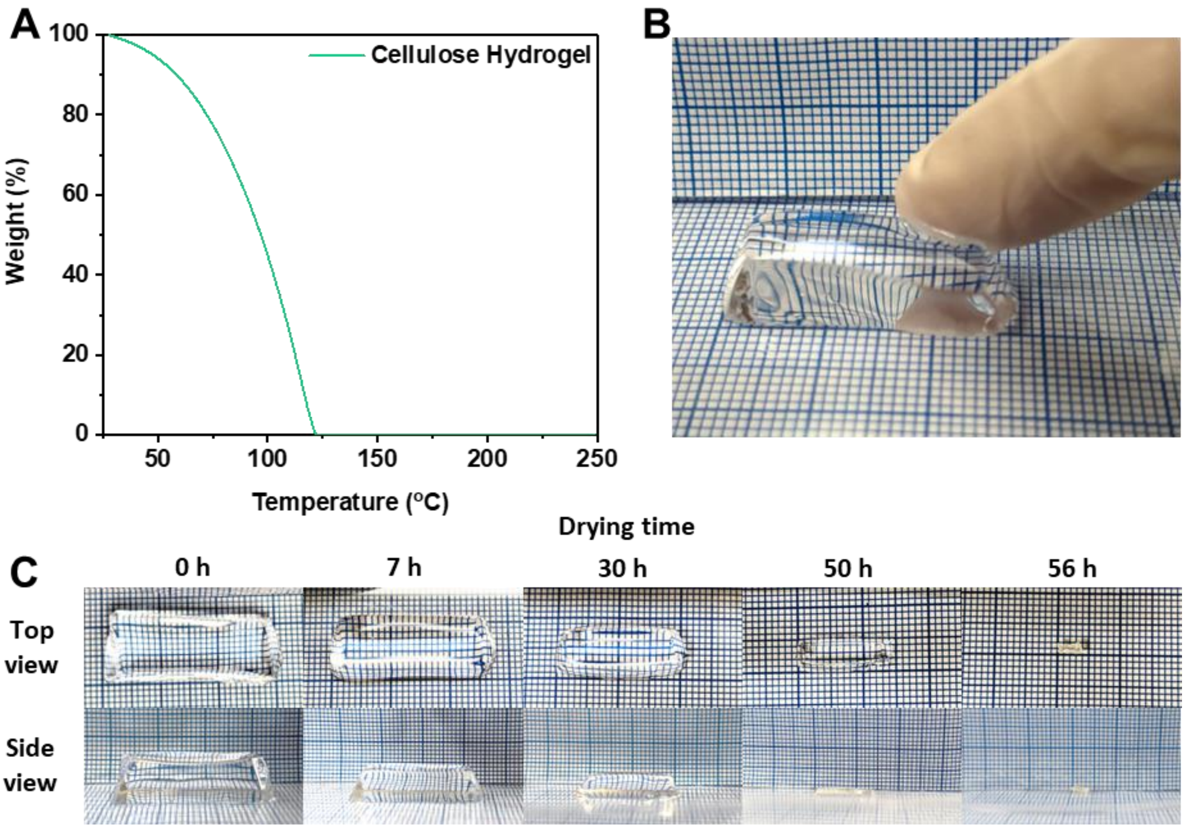

Figure S4. Cellulosic hydrogel's water content, drying time and shrinkage. (**A**) Thermogravimetric curve indicating a very high water content of around 99.9 wt%. (**B**) Image shows a large deformation with small pressure applied on the hydrogel, demonstrating its softness. (**C**) Images shows the drying process of the hydrogel.

The cellulosic hydrogel was synthesized using a conventional method crosslinking carboxymethyl cellulose (CMC) with epichlorohydrin (ECH).[1,2] Briefly, 3wt % CMC was dissolved in alkaline solution (6 wt% NaOH in water). 30 g of the CMC solution reacted with 3ml of epicholorohydrin at 60 °C for 12 h. The hydrogel was then washed with DI water until pH reaches around 7. Due to the large amount of water present in the hydrogel, it exhibits a very high softness unsuitable for the hydroshaping process. The high water content also leads to a lengthy drying time along with an immense shrinkage upon drying. These features set hydrogels apart from hydroplastics.

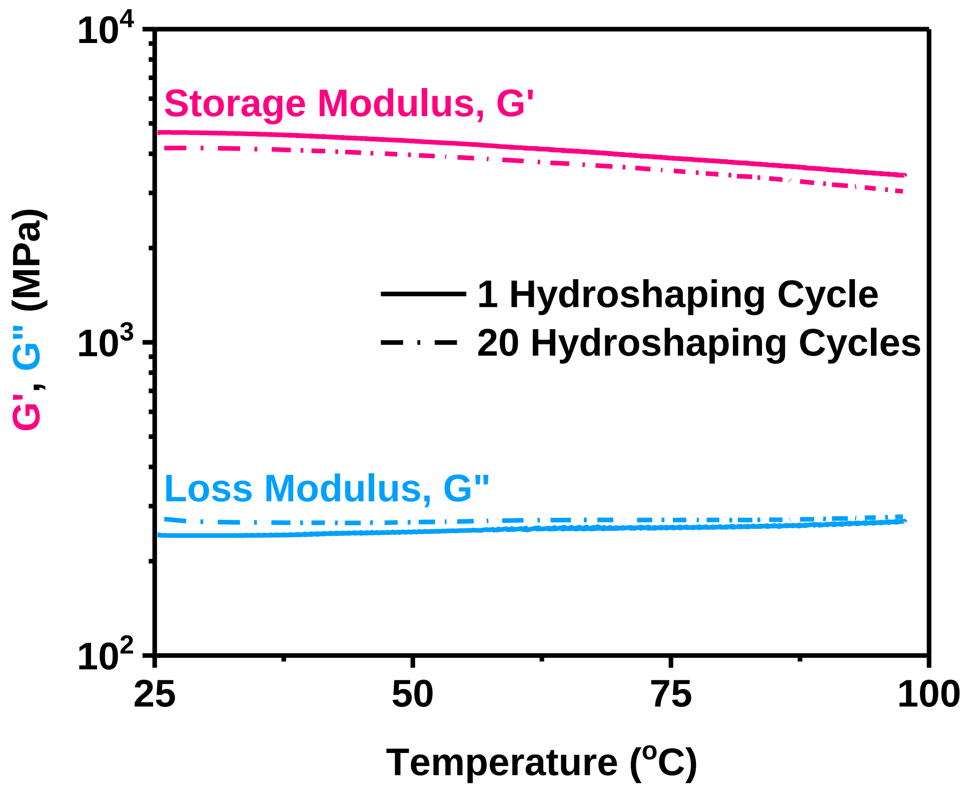

Figure S5. Dynamic mechanical analysis temperature sweep measurement of cellulose hydroplastic.

Measurement indicates that cellulose hydroplastic has similar mechanical performance after 1 hydroshaping cycle and 20 hydroshaping cycles.

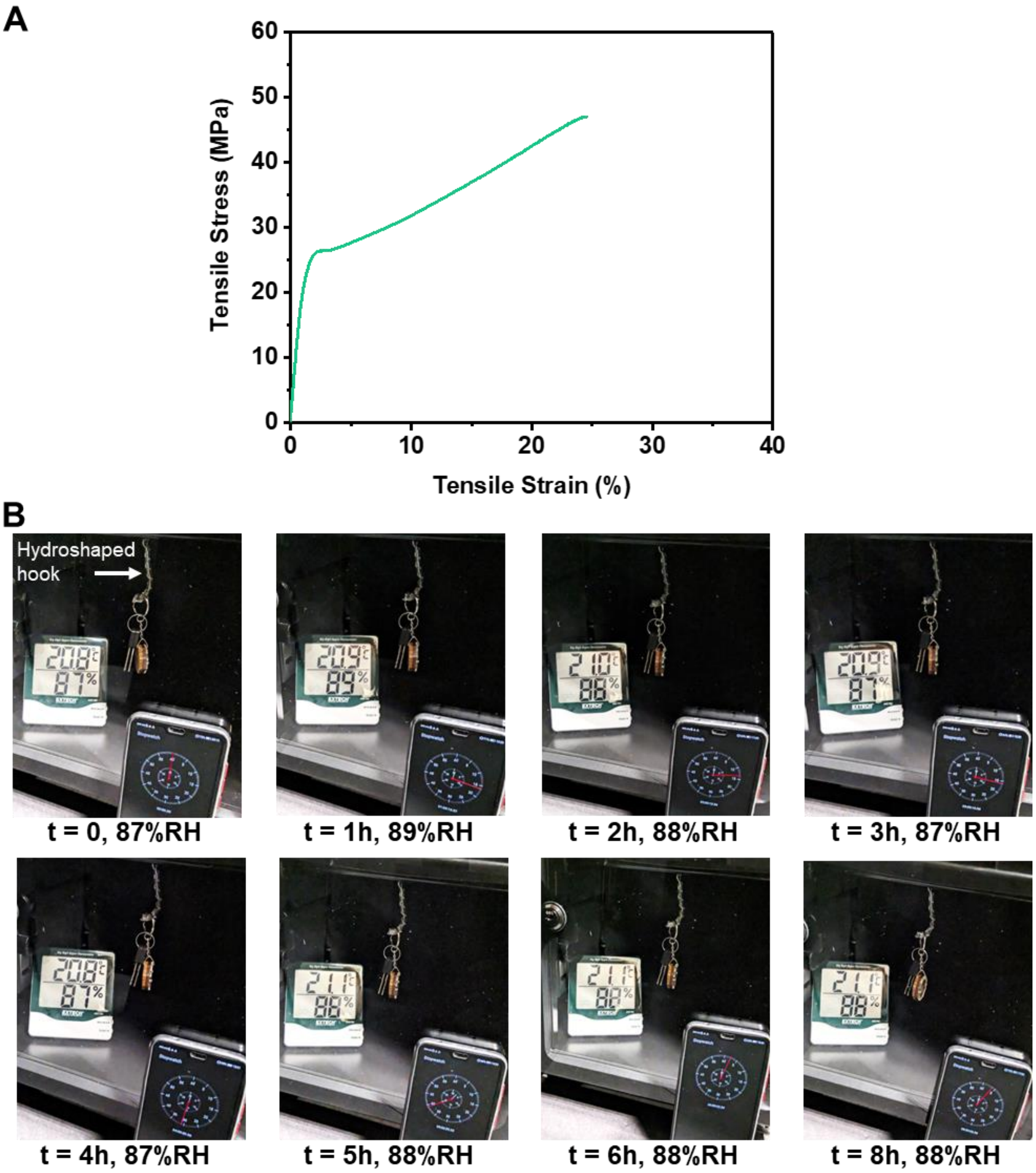

Figure S6. Structural stability of cellulose hydroplastic in high humidity environment. (**A**) Representative stress-strain curve of cellulose hydroplastic at 90%RH. (**B**) Images of a dry cellulose hydroplastic shaped into a J-shaped hook holding onto a bunch of keys at 87-89%RH for 8 h, without compromising its structural and geometrical integrity.

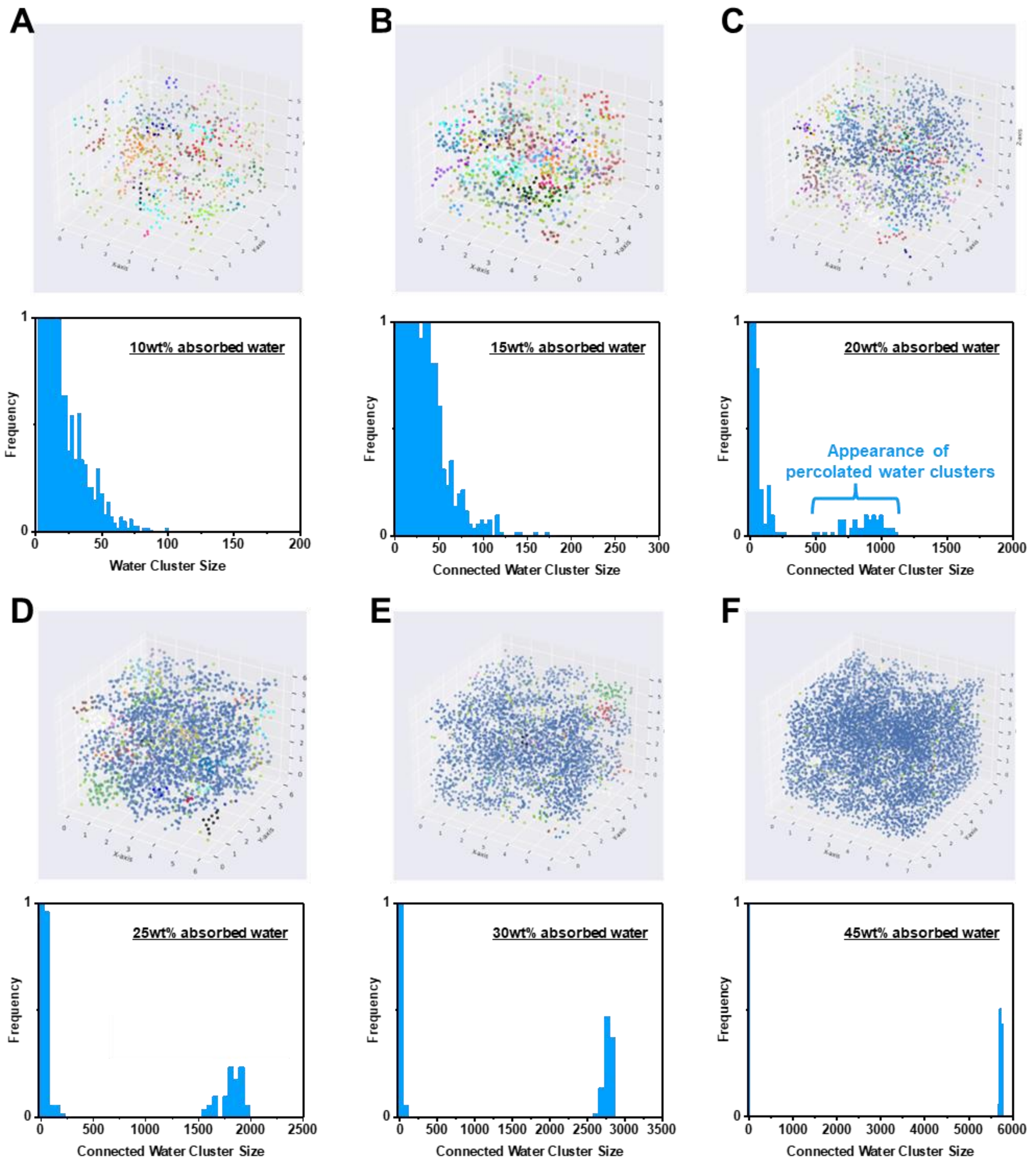

Figure S7. Water cluster analysis of amorphous cellulose with different water content. (**A**) 10 wt% water content. (**B**) 15 wt% water content. (**C**) 20 wt% water content. (**D**) 25 wt% water content. (**E**) 30 wt% water content. (**F**) 45 wt% water content.

Top images depict various water clusters differentiated by their color, with each dot representing a water molecule in cubic simulation box of equilibrated systems. The cluster analysis was performed by DBSCAN method[3] implemented on the 3D coordinates of oxygens of water in the simulation box. For cluster analysis, the optimum cut-off distance was set as 0.35 nm obtained by K-distance analysis and a minimum threshold of 3 connected water molecules was set to define a cluster core point. The histograms of shows the water cluster size distribution in respective systems. The results shown are the time-average values from last 10ns of simulation trajectory.

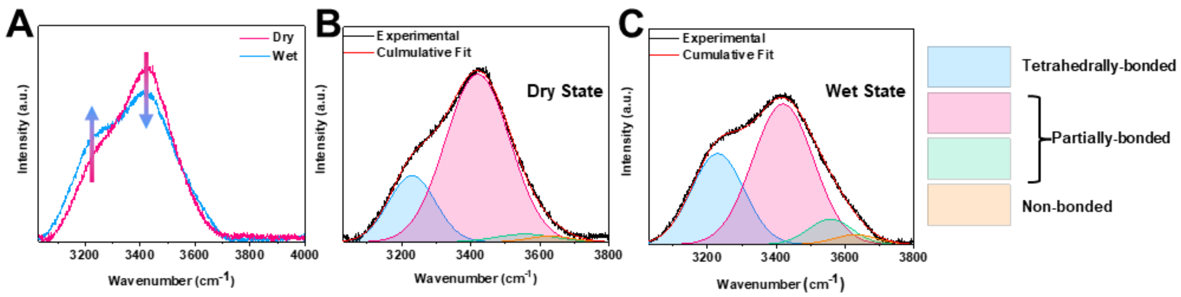

Figure S8. Deconvolution of Raman scattering spectroscopy O-H bond stretching band of cellulose hydroplastic. (**A**) Raman spectrum of dry state and wet state. (**B**) Deconvoluted dry state O-H bond stretching band. (**C**) Deconvoluted wet state O-H bond stretching band.

Transiting from the dry cellulose hydroplastic to the wet hydroplastic, a red-shift can be observed from the intensity increase at smaller wavenumber between 3100-3300 cm^{-1}, and intensity reduction at larger wavenumber between 3300-3500 cm^{-1} (**Figure S8A**). The O-H stretching band can be deconvoluted into 4 peaks centered at wavenumber 3230, 3420, 3557 and 3630 cm^{-1}, representing tetrahedrally-bonded water, partially-bonded water (both 3432 and 3557 cm^{-1}) and non-bonded water molecules, respectively. The dry state has areal proportion of 21.2%, 77.0% and 1.8%, for tetrahedrally-bonded water, partially-bonded water and non-bonded water molecules, respectively (**Figure S8B**). The wet state has areal proportion of 32.2%, 65.0% and 2.8%, for tetrahedrally-bonded water, partially-bonded water and non-bonded water molecules, respectively (**Figure S8C**). This corresponds to a significant increase in tetrahedrally-bonded water molecules and a reduction in partially-bonded water molecules upon transiting from dry to wet state.

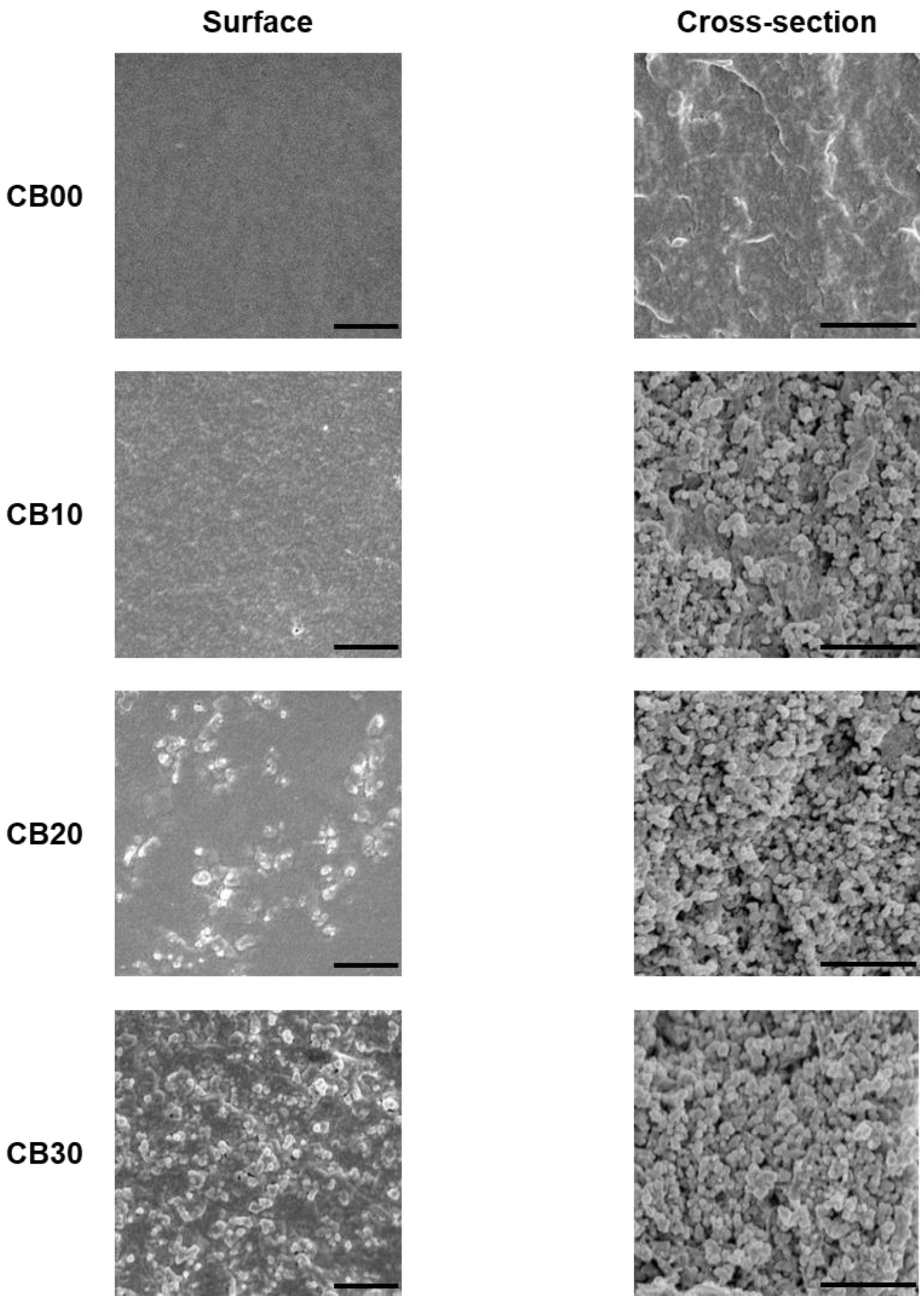

Figure S9. Surface and cross-sectional SEM images of cellulose hydroplastic and its composites. Scalebar: 1 µm.

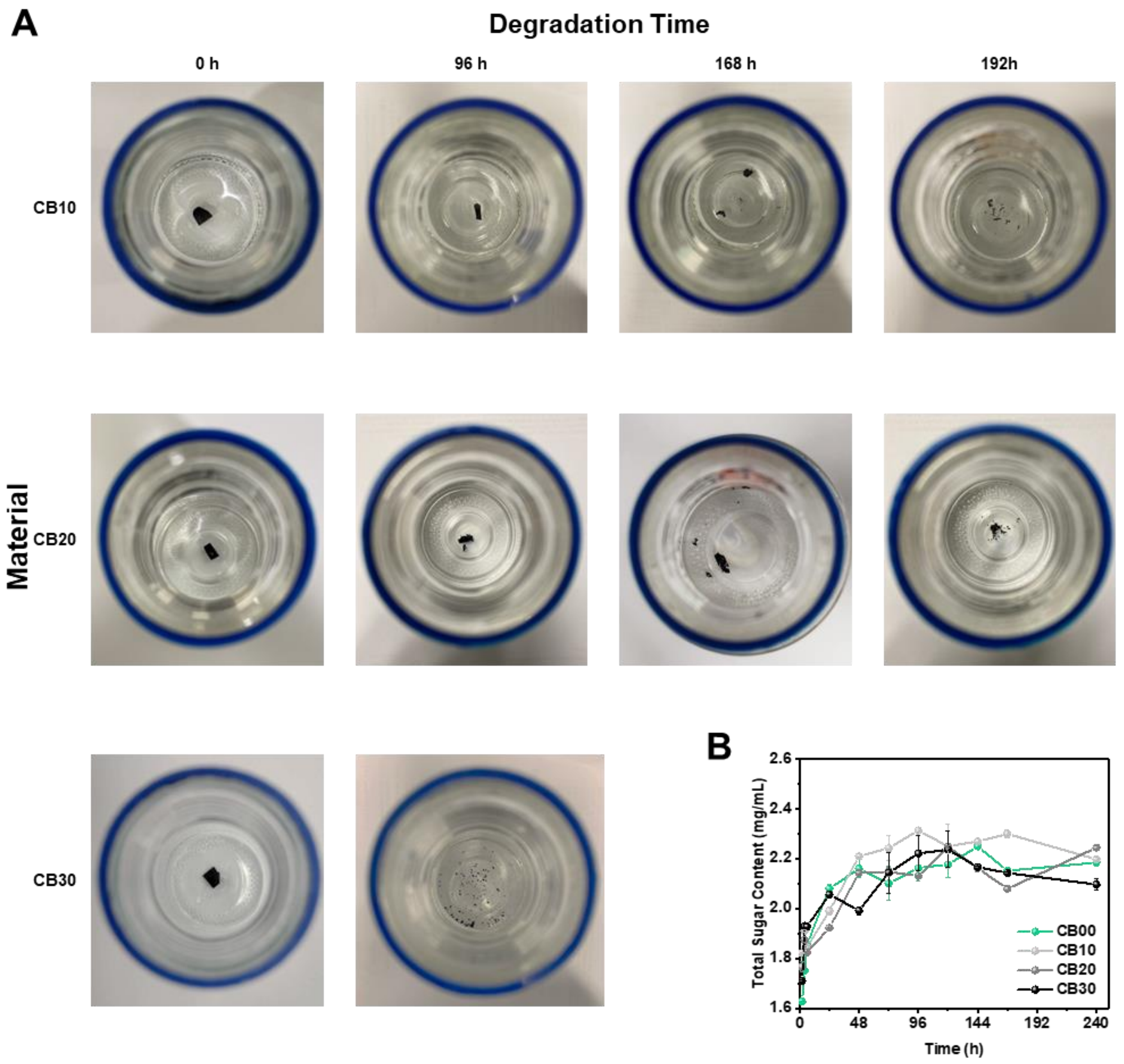

Figure S10. Biodegradation of cellulose-carbon hydroplastic composites. (**A**) Images showing the process of cellulose-carbon hydroplastic composites enzymatic biodegradation at different time intervals. (**B**) Total sugar content as a function of time in the enzymolysis solution at which the cellulose-carbon hydroplastic composites undergo enzymatic biodegradation.

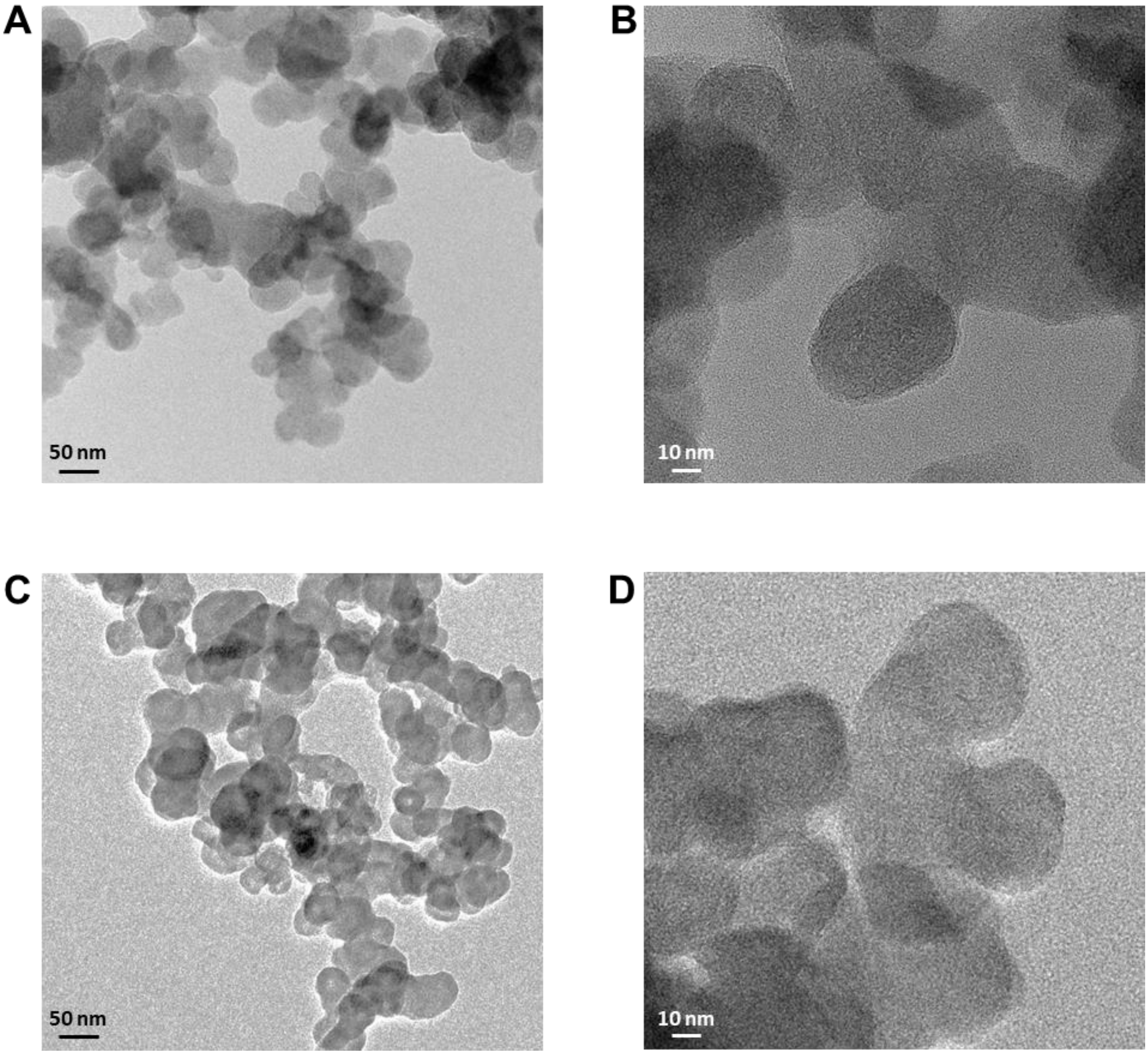

Figure S11. TEM images of carbon black nanoparticles. (**A–B**) Pristine. (**C–D**) Recycled after enzymatic degradation of cellulose-carbon hydroplastic composites.

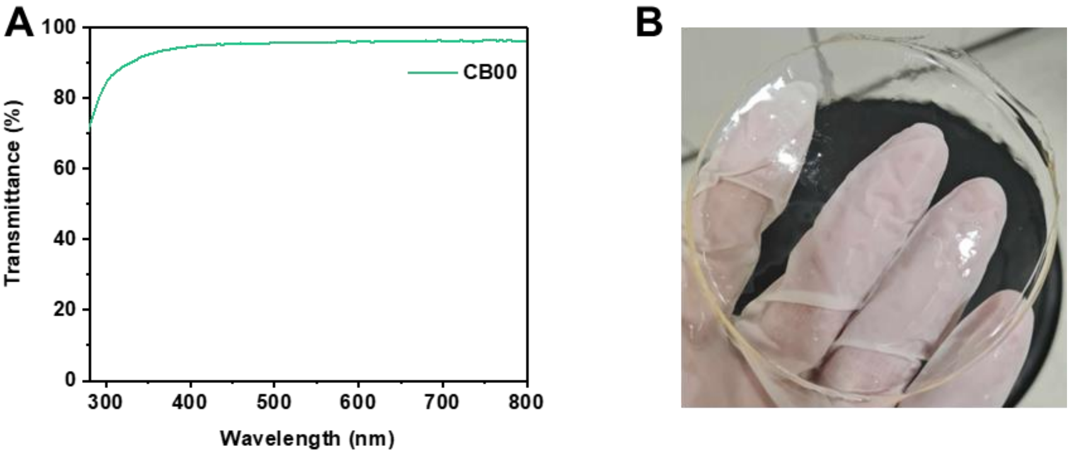

Figure S12. Transparency of cellulose hydroplastic. (**A**) Transmittance of cellulose between wavelength of 280-800 nm. (**B**) Image of transparent cellulose hydroplastic.

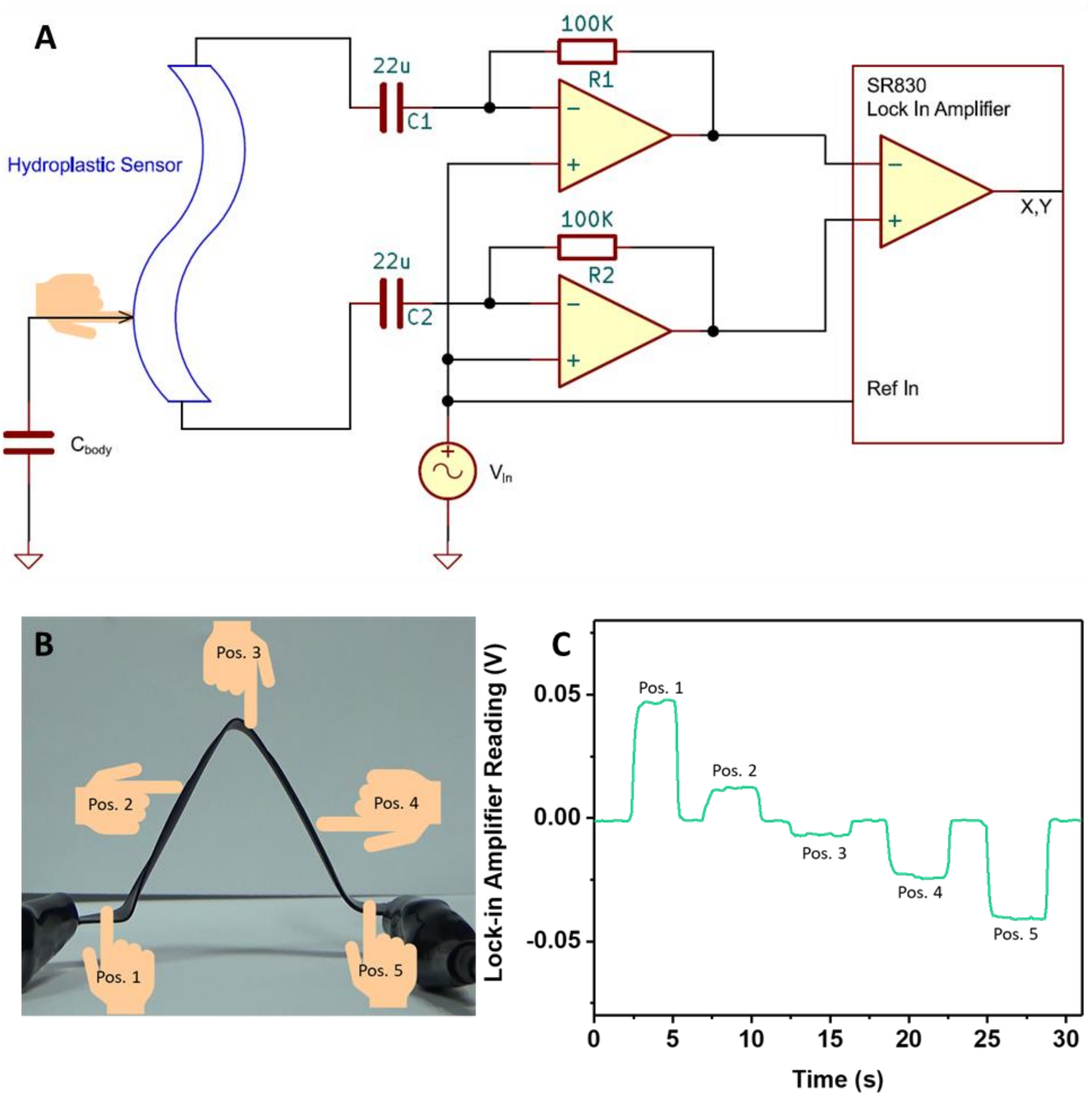

Figure S13. Surface capacitive sensor system of strip hydroplastic sensors. (**A**) Schematic of the signal conditioning circuit for touch position sensing. (**B**) Labelling of touch positions on "inverted-V-shaped" hydroplastic sensor. (**C**) Lock-in amplifier reading when various positions are touched as a function of time.

For the hydroshaped strip sensor, the body impedance (Z_{body}) at kHz frequencies is largely capacitive and can be modelled as a 100 pF capacitor (C_{body}) in **Fig. S13A**. This impedance is connected to the hydroplastic sensor (total resistance $2R_{hydroplas}$) at the touch point, which divides the hydroplastic sensor into two resistors with values $R_{hydroplas} + x$ and $R_{hydroplas} - x$. x is proportional to the displacement of the touch point from the centre of the hydroplastic sensor, and $x = 0$ would correspond to a touch exactly in the middle of the linear sensor.

The op-amps apply an AC voltage of $v_{in} = V_{in} \cos \omega t$ to the sensor, and when a touch happens, the body capacitance causes some AC current ($i_1 + i_2$) to flow into the sensor. Importantly, any asymmetry due to the touch point ($x \neq 0$) results in a slightly different AC current through the two op amp feedback resistors. The currents through the feedback resistors are:

$$i_1 = \frac{v_{in} - v_t}{R_{hydroplas} - x}$$

$$i_2 = \frac{v_{in} - v_t}{R_{hydroplas} + x}$$

Here, $v_t = Z_{body}(i_1 + i_2)$ is the AC voltage at the touch point. current difference can be solved,

$$i_2 - i_1 \approx \frac{v_{in}x}{Z_{body}R_{hydroplas}}(1 - \frac{R_{hydroplas}^2 - x^2}{2Z_{body}R_{hydroplas}} + \cdots)$$

To first order in x (this is justified because $Z_{body} \gg R_{hydroplas}$ and x), the output AC voltage measured by the lock in amplifier is

$$v_{sig} = R_{fb}(i_2 - i_1) \approx \frac{R_{fb}v_{in}x}{Z_{body}R_{hydroplas}}$$

Thus, the voltage measured by the lock in amplifier is proportional to the displacement x. In particular, since the body impedance is largely capacitive, the imaginary part of v_{sig} dominates, and we can define

$$v_{Lock-in} \equiv Im(v_{sig})$$

In the actual implementation of the circuit, a 100 kΩ resistor is used for R_{FB}, and the hydroplastic sensor is AC coupled to the op-amps with 22 µF capacitors. This is to eliminate the effect of unequal input offset voltages of the op-amps. v_{in} is a 1 Vpp signal at 1 kHz supplied by a SR830, and the signals from the two op amp outputs goes into the SR830 in differential input mode which directly measures the complex phasor v_{sig}. Because the body impedance is largely capacitive, the quadrature output of the lock in amplifier, $v_{Lock-in} \equiv Im(v_{sig})$ is obtained by using the Y-output of the SR830 and setting the internal phase shift of the lock-in amplifier to 0. **Figure S13B–C** shows this lock in amplifier reading which varies as different points on the hydroplastic sensor is touched.

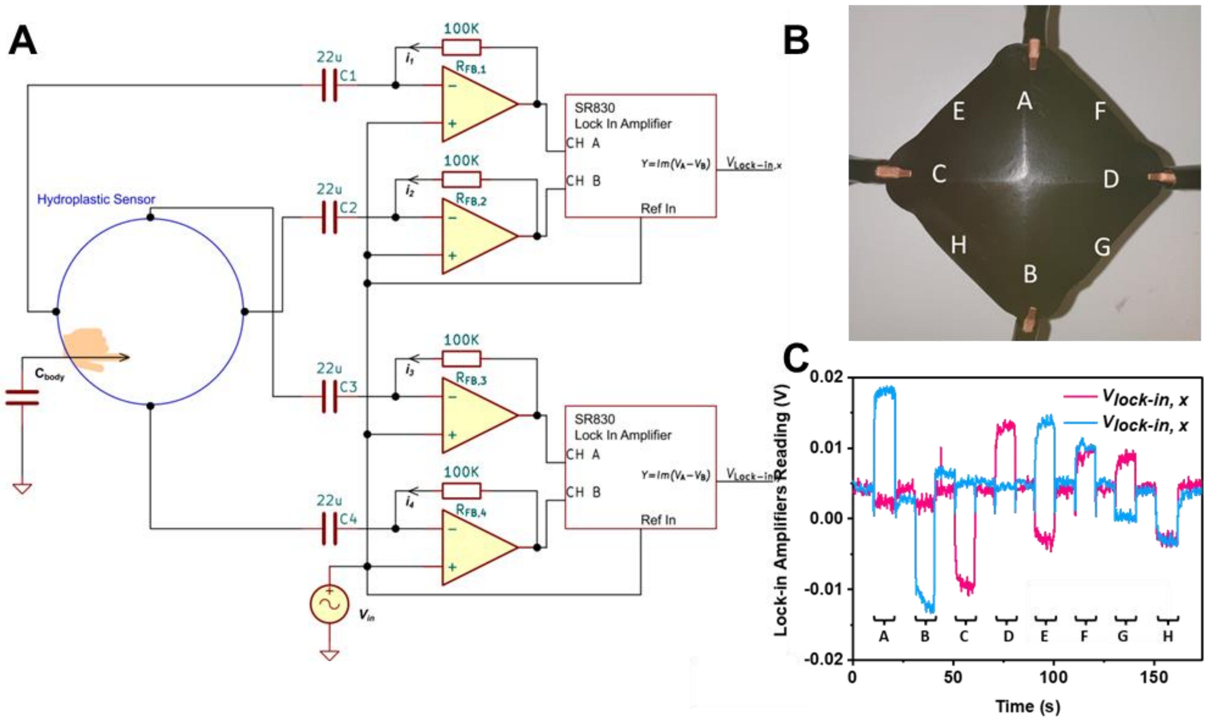

Figure S14. Surface capacitive sensor system of four quadrant dome-shaped hydroplastic sensor. (**A**) Schematic of the signal conditioning circuit for 2D touch position sensing. Two independent lock-in amplifier channels allow for 2D sensing of the touch position. (**B**) Labelling of touch positions on dome-shaped 2D hydroplastic sensor. (**C**) Lock-in amplifier readings when various positions are touched as a function of time.

The same principle described for **Figure S13** can be extended to the dome-shaped touch sensor. **Figure S14** shows how two independent amplifier channels connected to a single hydroplastic sensor can enable two-dimensional touch positional sensing.[4] One SR830 lock-in amplifier measures the x-position, while the other measures the y-position. By reading these values in real-time simultaneously, the 2D position of the touch point can be measured.

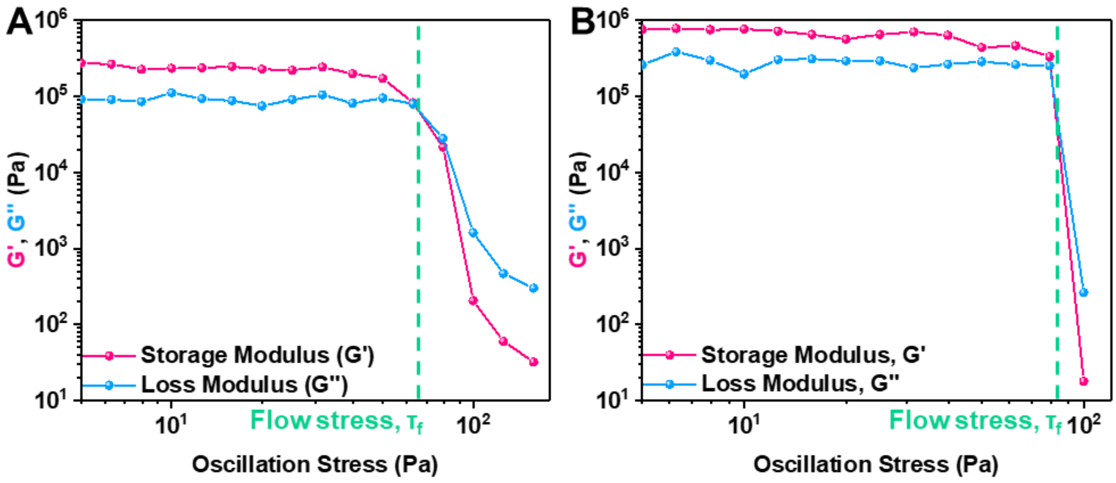

Figure S15. Rheological amplitude sweeps of 3D-printing inks. (**A**) Conductive CB30 ink. (**B**) Non-conductive CB00 ink.

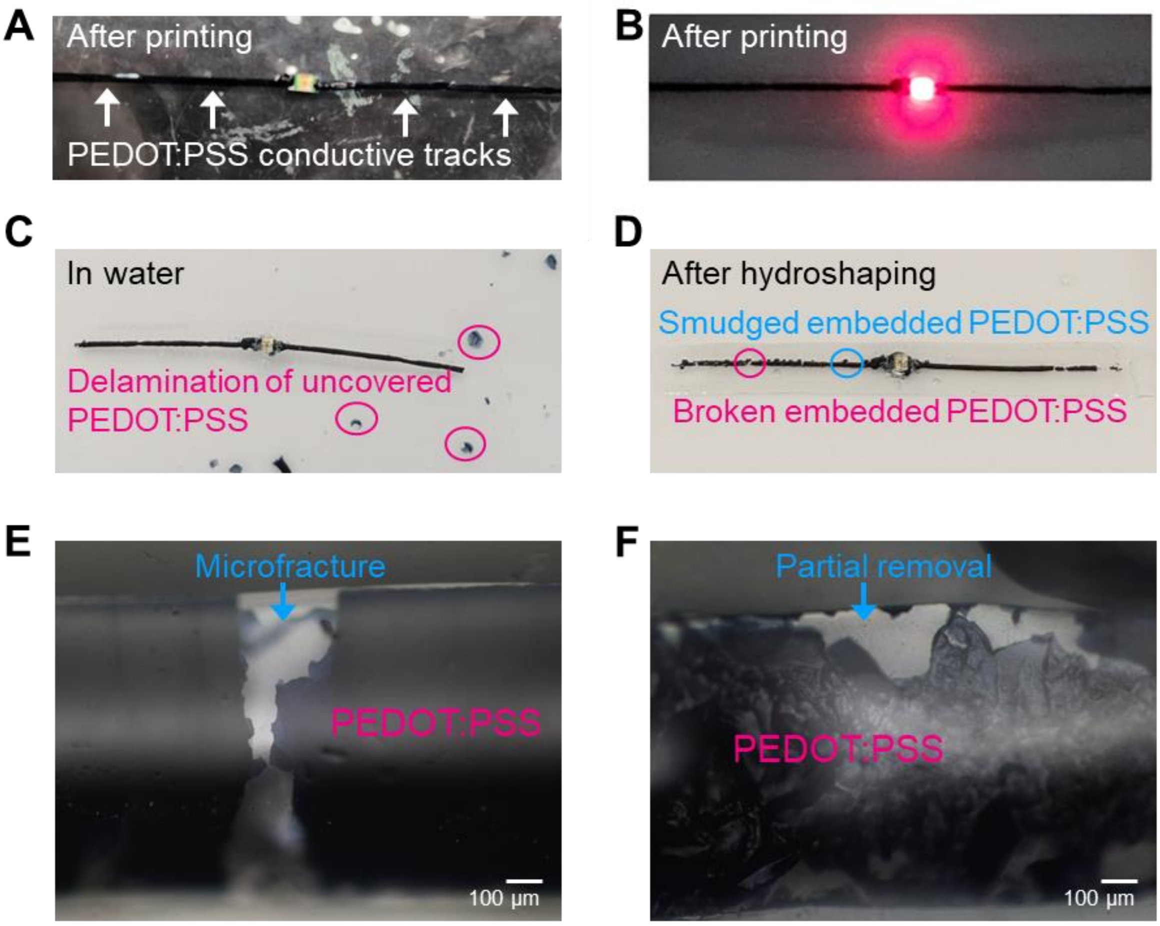

Figure S16. 3D-printed PCB with PEDOT:PSS. (**A**) LED placed between two well-printed PEDOT:PSS conductive tracks on cellulose acetate. (**B**) Lighting up of the LED when current is passed through the two ends of the printed PEDOT:PSS tracks. (**C**) Delamination of uncovered PEDOT:PSS tracks from cellulose hydroplastic in water. (**D**) Defects on the printed PEDOT:PSS after the hydroshaping process. (**E**) Optical microscope image showing microcracks along a printed PEDOT:PSS track after hydroshaping. (**F**) Optical microscope image showing the partial removal of some PEDOT:PSS from its original location after hydroshaping.

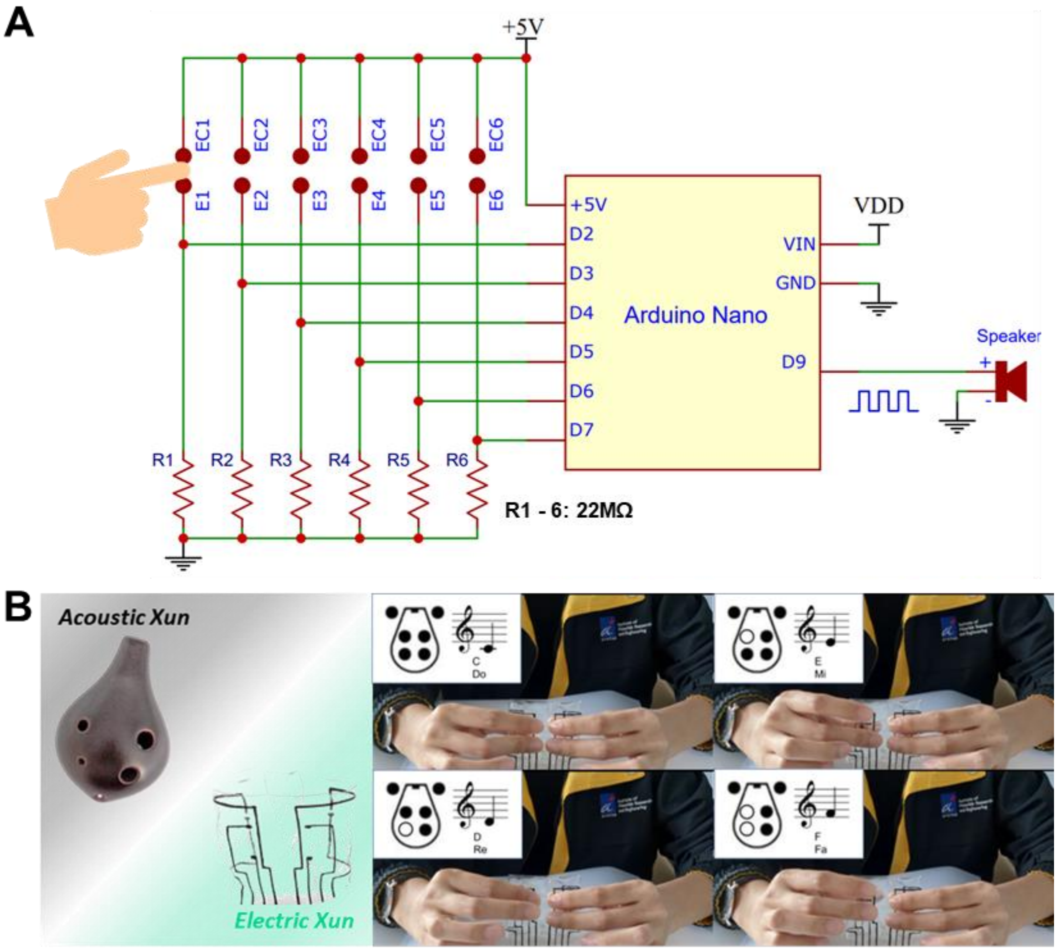

Figure S17. Schematic circuit of Electric *Xun* system. (**A**) Schematic circuit diagram of hydroshaped electric *Xun*. (**B**) Illustration of Acoustic Xun versus electric *Xun* and demonstration of playing the electric *Xun*.

The system consists of the 3D-printed hydroplastic electric *Xun* sensor, Arduino Nano chipset and a speaker. The *Xun* possesses an array of six touch- pads, each of which comprises an electrode contact pair. Within each electrode pair, the first electrode (EC1 - 6) is connected to +5V in common with those of the others, while the second electrode (E1 - 6) is weakly pulled to ground via a high ohmic (22 MΩ) resistor. Each of the second electrode is also coupled to digital GPIOs (D2 - D7) of Arduino Nano chipset configured as input pins for detecting the digital states. When the touch pads are not pressed, the electrode pair contacts are opened by default such that the interfacing GPIO pins normally assume digital low states. As the player presses a touch pad, the electrode pair contact is electrically closed by the fingers' skin impedance. This pulls up the electrical potential at the interfacing GPIO pin inducing a digital high state. Based on the combination of the input states detected, the microcontroller is programmed to generate pulse signal of the specified frequencies at the digital output pin, D9, thus driving a low-power audio speaker to produce the desired musical note in response to the user's fingering. The musical notes and acoustic signal frequencies generated in response to each touch combinations are presented in **Table S2**.

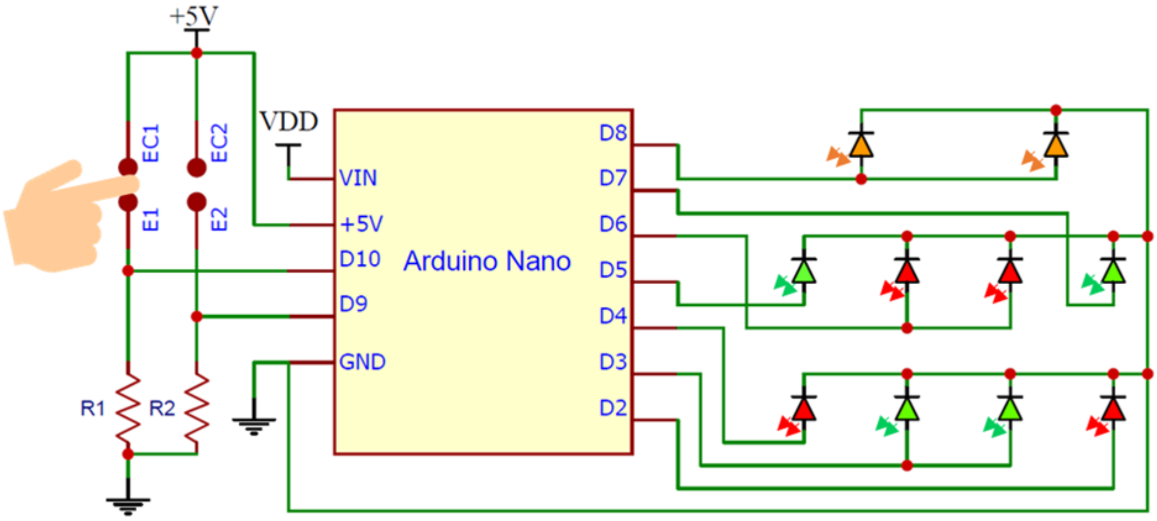

Figure S18. Schematic circuit of 3D-printed hydroshapable interactive display.

The system consists of a 3D-printed hydroplastic composite with conductive tracks (CB30), embedded LEDs and a microcontroller. A pair of resistive buttons, to detect the input from the user, is formed by having a 1 mm gap between two disconnected conductive tracks. The microcontroller is programmed to detect the touch sensors' input and power up the selected LEDs for displaying the desired response by the user.

The LEDs are connected to digital GPIOs (D2-D8) of an Arduino Nano microcontroller programmed as the output pins to turn on the selected LEDs. The resistive buttons comprise of printed electrode contact pairs (E1/EC1 and E2/EC2). For each electrode pair, the first electrode (EC1 - 2) is connected to +5V, while the second electrode (E1 - 2) is normally grounded via a high ohmic (22 MΩ) resistor. The second electrode is also coupled to digital GPIOs (D9 – D10) configured as the input pins. As the user presses any of the resistive buttons, the selected electrode pair contact is electrically closed by the fingers' skin impedance, thus transiting the respective detecting pins to digital high state. The microcontroller then turns on the selected LEDs that represents the response of the user.

Table S1. Mechanical properties of cellulose-carbon composites

**Material**	**Young's Modulus (GPa)**	**Tensile Strength (MPa)**	**Elongation at break (%)**
CB00	6.83 ± 0.89	64.8 ± 9.5	20.3 ± 2.1
CB10	8.22 ± 0.39	65.2 ± 8.2	20.7 ± 5.4
CB20	10.45 ± 0.52	59.7 ± 2.5	19.1 ± 1.6
CB30	13.74 ± 0.75	43.1 ± 7.1	11.3 ± 3.2

Table S2. Musical notes and acoustic signal frequencies generated in response to different digital buttons combinations of electric *Xun*.

Musical Note	Frequency (Hz)	Input Pins Triggered
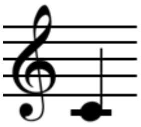<br>C<br>Do	**262**	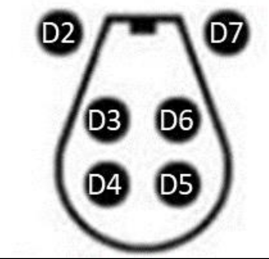
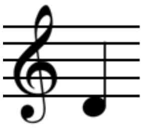<br>D<br>Re	**294**	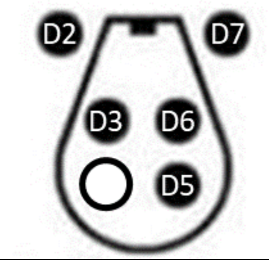
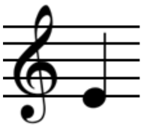<br>E<br>Mi	**330**	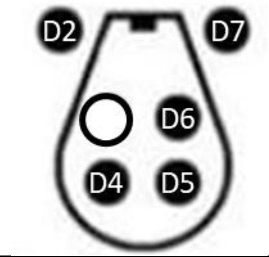
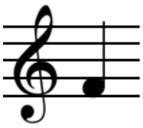<br>F<br>Fa	**349**	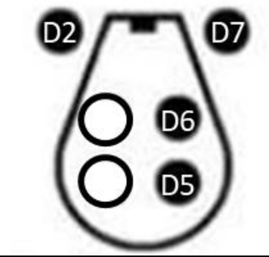
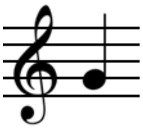<br>G<br>So	**392**	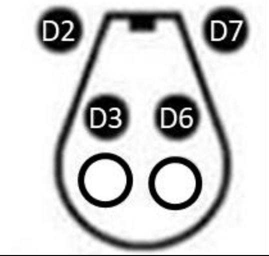
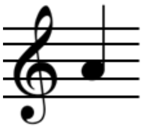<br>A<br>La	**440**	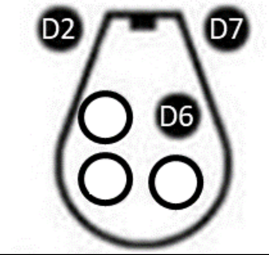

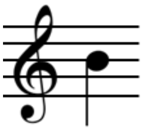 B<br>Ti	**494**	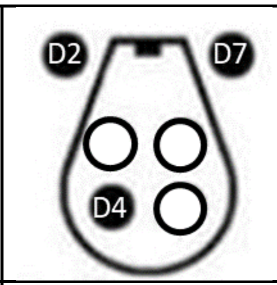
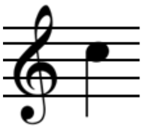 High C<br>High Do	**523**	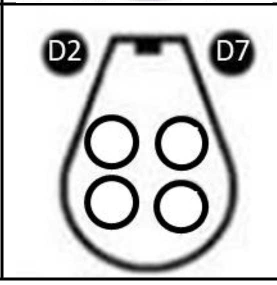

References

[1] D. Jeong, S.-W. Joo, Y. Hu, V. V. Shinde, E. Cho, S. Jung, *Eur. Polym. J.* **2018**, *105*, 17.

[2] C. Chang, B. Duan, J. Cai, L. Zhang, *Eur. Polym. J.* **2010**, *46*, 92.

[3] M. Ester, H.-P. Kriegel, J. Sander, X. Xu, in *Proc. Second Int. Conf. Knowl. Discov. Data Min.*, AAAI Press, **1996**, pp. 226–231.

[4] Y. Cao, Y. J. Tan, S. Li, W. W. Lee, H. Guo, Y. Cai, C. Wang, B. C.-K. Tee, *Nat. Electron.* **2019**, *2*, 75.
